# Lily Cultivars Have Allelopathic Potential in Controlling *Orobanche aegyptiaca* Persoon

**DOI:** 10.1371/journal.pone.0142811

**Published:** 2015-11-13

**Authors:** Min Chai, Xiaopei Zhu, Hongxia Cui, Chuangdao Jiang, Jinzheng Zhang, Lei Shi

**Affiliations:** 1 Key Laboratory of Plant Resources and Beijing Botanical Garden, Institute of Botany, Chinese Academy of Sciences, Beijing 100093, China; 2 University of Chinese Academy of Sciences, Beijing 100049, China; Northwest A&F University, CHINA

## Abstract

As a devastating holoparasitic weed, *Orobanche aegyptiaca* Persoon. (Egyptian broomrape) causes serious damage to agricultural production and threatens economic development, which has raised widespread concern. The present study was conducted to determine whether lilies have the potential to be used as ‘trap crops’ for controlling *O*. *aegyptiaca* Persoon. In the experiments, the ability of three popular lily cultivars (*Lilium* Oriental hybrids ‘Sorbonne’, *Lilium* LA (Longiflorum hybrids x Asiatic hybrids) hybrids ‘Ceb Dazzle’, and *Lilium* Longiflorum hybrids (*L*. *formosanum* x *L*. *longiflorum*) ‘*L*. *formolongo*’) to induce *O*. *aegyptiaca* Persoon. seed germination was assessed. Parts of the three lily cultivars, including the rhizosphere soil and underground and above-ground organs, all induced “suicidal germination” of parasitic *O*. *aegyptiaca* Persoon. seed at four growth stages. Specifically, Sorbonne and Ceb Dazzle behaved with similar allelopathy, and the bulb, scale leaf and aerial stem exhibited stronger allelopathic effects on *O*. *aegyptiaca* Pers. germination compared to other organs. Aqueous L. *formolongo* leaf extracts may contain more stable, effective stimulants given that they induced the highest germination rate at 76.7% even though the extracts were serially diluted. We speculate that these organs may be advantageous in further isolating and purifying economical active substances that can be substitutes for GR24. These results indicate that lilies have the potential to be used as a trap crops or can be processed into green herbicide formulations that can be applied in agriculture production to rapidly deplete the seed bank of *O*. *aegyptiaca* Persoon. parasitic weeds in soil.

## Introduction

*Orobanche* spp. (broomrapes), a type of widely distributed holoparasitic weed found throughout the Mediterranean region, Western Asia and Eastern Europe, causes enormous economic loss by parasitizing primarily on Solanaceae, Cucurbitaceae, Leguminosae, Compositae, Cruciferae Apiaceae and Gramineae plants for nutrients and water [1–3]. Of the diverse *Orobanch* spp. species, *O*. *aegyptiaca* Persoon., *O*. *cernua* Loefl., *O*. *crenata* Forsk., *O*. *Cumana* Wallr., *O*. *minor* Sm. and *O*. *ramosa* L. have a wide parasitizing spectrum and are capable of doing more harm than any of the other *Orobanche* spp. species [3, 4].

A large reserve of *Orobanche* spp. seeds exist in infected soils, and it is too labor intensive and ineffective to reduce the seed bank using mechanical, manual or pesticide application [5–7]. Controlling parasitic weeds with trap crops may be a biologically promising and practical method in farming. Trap crops are non-host species whose roots exude chemical stimulants required for parasite germination without haustoria formation [3, 8]. To date, many efforts have been implemented to screen various trap crops for use against parasitic weeds. For example, wheat, maize, rice, cotton, soybean and rape have been identified as trap crops in controlling *Orobanche* spp. [9–14].

Researchers have isolated and identified the exudates produced and released by the roots of host and some non-hosts plants, such as tomato, sorghum, maize, pea and Arabidopsis. Secondary metabolites called strigolactones (SLs) have been confirmed as the major class of stimulants that induce parasitic *Orobanche* and *Striga* spp. seed germination [15–19]. In addition, many studies have shown that field applications of SLs or synthetic SL analogue stimulants, such as GR24, GR3 and Nijmegen1, provide partial control of *Orobanche* spp. due to instability of the compound, particularly under alkaline conditions [20–24]. And now GR24 is usually used as a general standard in germination assays, which elicits >60% germination at 100 nM. Therefore, it is necessary to select more potential trap crops and stable allelopathic substances from non-hosts of *Orobanche* spp., which contribute to the synthesis of more effective germination stimulants such as SLs. A good focus of allelopathy has been done on grain, vegetable crops and traditional medicinal herbs for *Orobanche* spp., while few studies have been conducted on the allelopathic effects of ornamental plants for *Orobanche* spp. [12–14, 25, 26].

Due to abundant bioactive constituents in bulbs, lilies are popular ornamental plants that are also used as medicinal herbs in China for treatment of various human and animal diseases [27, 28]. Significant allelopathic effects caused by unknown secondary metabolites have been shown to be key factors that lead to continuous cropping obstacles in lilies [29–33]. However, it is unknown whether *Orobanche* spp. germination inducers are available in lilies. The objective of this study was to compare extracts from three popular lily cultivars at various growth stages on inducement of *O*. *aegyptiaca* Pers. germination. This information can further expand applications in weed control based on ornamental and edible lily components.

## Materials and Methods

### Experiment Materials and Chemicals

Three popular lily cultivars, *Lilium* Oriental hybrids (some lily species were selected as parents in hybridized breeding) ‘Sorbonne’, *Lilium* LA (Longiflorum hybrids x Asiatic hybrids) hybrids ‘Ceb Dazzle’ and *Lilium* Longiflorum hybrids (*L*.*formosanum* x *L*.*longiflorum*) ‘*L*. *formolongo*’, were purchased from Beijing Sheng sitong Ecological Technology co., LTD at Changping district of Beijing. Pot experiments were conducted at the Institute of Botany, Chinese Academy of Science in Beijing, China in January 2014. Pots were placed in a greenhouse at an approximate temperature of 25°C with daily sunlight. Pots were watered as necessary and kept moist. Experimental soil was mixed using peat and sand at a ratio of 2:1 with a pH of 6.83. Two bulbs of each lily cultivar were planted in a plastic pot (15 cm high by 20 cm diameter) containing 1.5 kg of soil.

*O*. *aegyptiaca* Pers. seeds collecting in 2010 from infested tomato (*L*. *esculentum* Mill) fields in Xinjiang Uygur Autonomous Region, China and the synthetic strigolactone GR24 were supplied by Professor Yongqing Ma in 2013 (Institute of Soil and Water Conservation and Northwest Agriculture & Forestry University, Chinese Academy of Sciences, Yangling, Shanxi, China). The seeds were stored by a sealed plastic tube and placed in a cool, dry and dark environment. The GR24 were stored in the refrigerator at -20°C.

*O*. *aegyptiaca* Pers. seeds were surface-sterilized in 1% sodium hypochlorite (v/v) for 3 min and thoroughly rinsed with sterile distilled water. Approximately 30 air-dried *O*. *aegyptiaca* Pers. seeds were sown on each glass fiber disk Glass (5 mm diameter) which was arranged evenly on the double moist filter paper placed in the 7 cm Petri dishes. Then 1 mL of the distilled water as ‘conditioning medium’ was added into Petri dishes, making the seeds to touch a wet surface without soaking. Finally, sealed Petri dishes were incubated in the dark at 25°C for conditioning period of 2 days and then used in germination bioassays [25].

### Collection of Lily Rhizosphere Soils and Plant Samples

Rhizosphere soils and lily plant samples were collected at four developmental stages (sprouting, the terminal bud began to sprout and grow to about 10–15 cm; leaf expansion, the flower bud was forming and the stem grown to about 40–45 cm; flowering, most of flower buds was blooming; and increasing bulblet weight, about 3 weeks after all the flowers withered). At each samples-taking stage, three pots per cultivar were picked out as three individual repetitions, at the same time, both lily plants in each pot have been divided into six tested parts respectively and the same tested parts between them were mixed as one sample.

Specifically, the lily’s root plus adhering soil carefully picked out of the nonrhizosphere soil. The loosely held soil was gently shaken off the roots, which was referred to as rhizosphere soil [34]. And the whole lily plant was divided into six tested parts, including the aerial stem, leaf and prop root belonging to above-ground organs and the bulb, scale leaf and roots as underground components. Each tested part rinsed thoroughly with water was shade-dried and then milled to pass through a 0.45 mm sieve. The final powder collected as tested part sample was stored at 0–5°C for further tests as described below, and when the power was extracted, each treatment was replicated three times.

### Preparation of Aqueous and Methanol Extracts

#### Aqueous Extracts

Rhizosphere soil sample collected from pot-cultivated lilies (5 g) plus 10 mL distilled water were ultrasonic treated for 30 min and then filtered. The filtered solutions are hereafter referred to as the undiluted rhizosphere soil extracts. Lily powder (0.1 g) sample made from above- and underground components was placed into a centrifuge tube (1.5 mL) and mixed with distilled water. The mixture was treated by ultrasound for 30 min and centrifuged at 6400 rpm for 2 min. The supernatant obtained was collected as undiluted plant sample extracts. These undiluted extracts were further diluted 10-, 100- and 1000-fold with distilled water. A total of 30 μL of each extract was added to *O*. *aegyptiaca* Pers. seeds on glass fiber disks in Petri dishes. Each treatment was replicated three times. Sealed Petri dishes were incubated in the dark at 25°C for 7 d, and the germination rate was examined with a microscope. Conditioned *O*. *aegyptiaca* Pers. seeds treated with GR24 and distilled water were used as positive and negative controls, respectively [35].

#### Methanol Extracts

Four concentrations of methanol extracts were obtained in the same manner as the aqueous extracts. In addition, 30 μL of the methanol extracts were added to an empty glass fiber filter disk without seeds and allowed to evaporate for 30 min at room temperature. A second glass fiber disk with conditioned *O*. *aegyptiaca* Pers. seeds was placed on top of the empty glass fiber disk. Autoclaved distilled water (30 μL) was added to the double glass fiber disks. Each treatment was replicated three times. Conditioned *O*. *aegyptiaca* Pers. seeds treated with GR24 and methanol were used as positive and negative controls, respectively. Remaining test procedures were similar to those described above [35].

### Statistical Analyses

Data were processed using Excel 2007, SPSS17.0 and 3.1.2 R software. Duncan's multiple range test was used to separate the means. Treatment means were compared using least significant difference tests at a 5% level of probability.

## Results

Germination rates of *O*. *aegyptiaca* Pers. seeds increased up to 90% when treated with the positive control GR24, while germination did not occur in seeds treated with distilled water and methanol, indicating that the *O*. *aegyptiaca* Pers. seeds were viable. In addition, *O*. *aegyptiaca* Pers. showed no seed germination when treated with aqueous and methanol extracts of potting substrates without cultivating lily plant. It may also illustrates that allelochemicals secreted by lily root include obligate germination stimulants for *O*. *aegyptiaca* Pers. and also eliminate interference of soil condition.

### Induction of *O*. *aegyptiaca* Pers. Seed Germination at the Sprouting Stage

**Fig 1:**
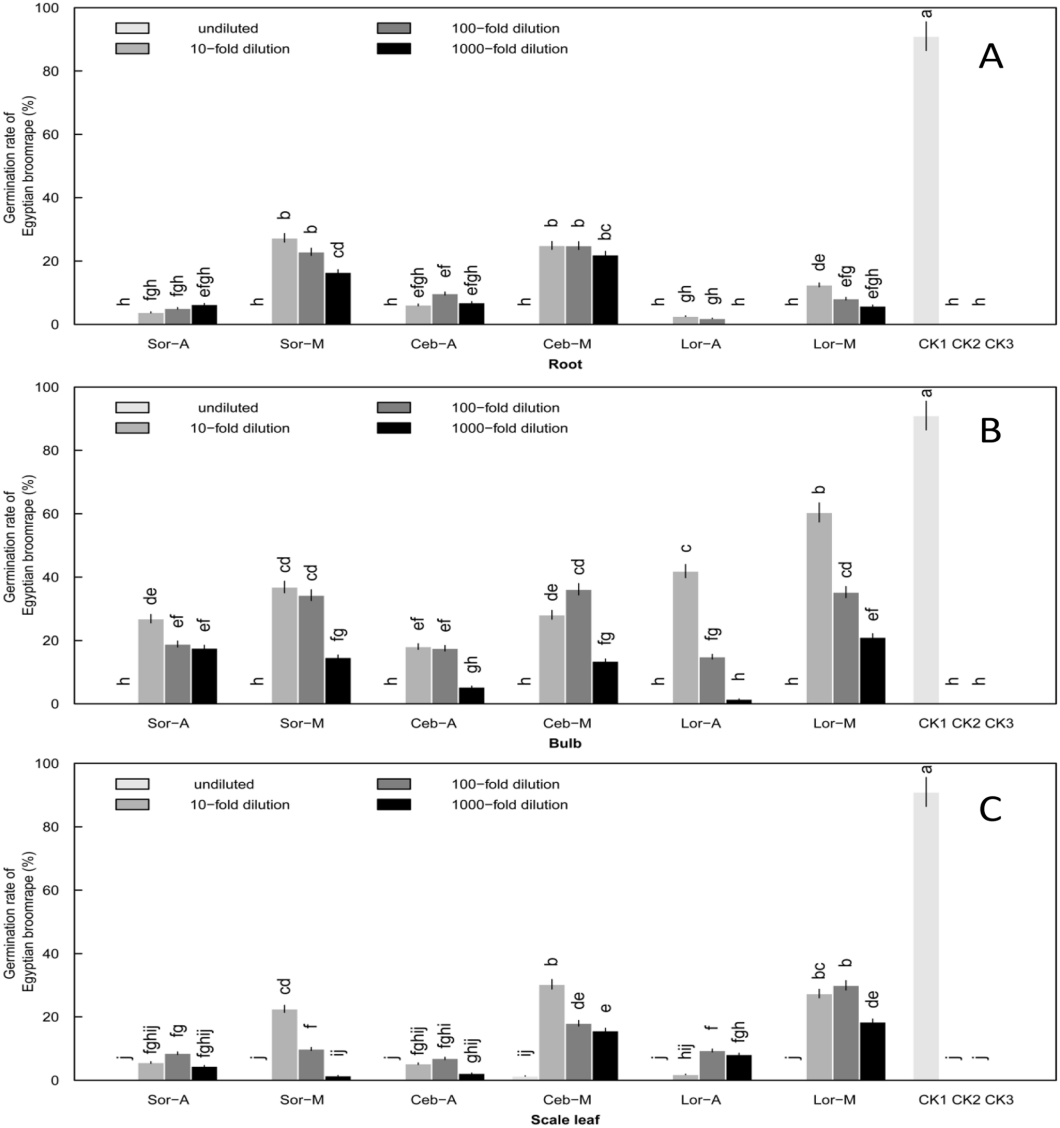
Induction of *O*. *aegyptiaca* Pers. germination by extracts of lily underground organs. Root (A), bulb (B) and scale leaf (C) at the sprouting stage. Lily underground organs were tested, including (A) root, (B) bulb and (C) scale leaf. Four concentrations of extracts were assessed: undiluted and 10-fold, 100-fold and 1000-fold dilutions. Abbreviations: Sor-A, Sorbonne aqueous extracts; Sor-M, Sorbonne methanol extracts; Ceb–A, Ceb Dazzle aqueous extracts; Ceb–M, Ceb Dazzle methanol extracts; Lor-A, *L*. *formolongo* aqueous extracts; Lor-M, *L*. *formolongo* methanol extracts; CK1, GR24 as positive control; CK2, Distilled water as negative control; CK3, Methanol as negative control. Error bars represent the standard error of the mean. Different small letters above the error bars indicate significant differences at 0.05 (ANOVA and Duncan's multiple range test). Matching letters in the same color column indicates that there were no significant differences between treatments.

At the sprouting stage, all three of the lily cultivars had broken dormancy with a short terminal bud. At terminal bud height about 10–15 cm, the bulb, scale leaf and root belonging to underground organs were selected for testing. *O*. *aegyptiaca* Pers. showed no germinated at all when treated with undiluted aqueous and methanol extracts. With increased extract dilutions, germination rates generally increased at first and then declined (Fig 1). The highest germination rates were induced by 10-fold or 100-fold dilution of aqueous and methanol extracts. Methanol extracts generally induced higher germination rates than aqueous extracts (S1 Table).

**Fig 2:**
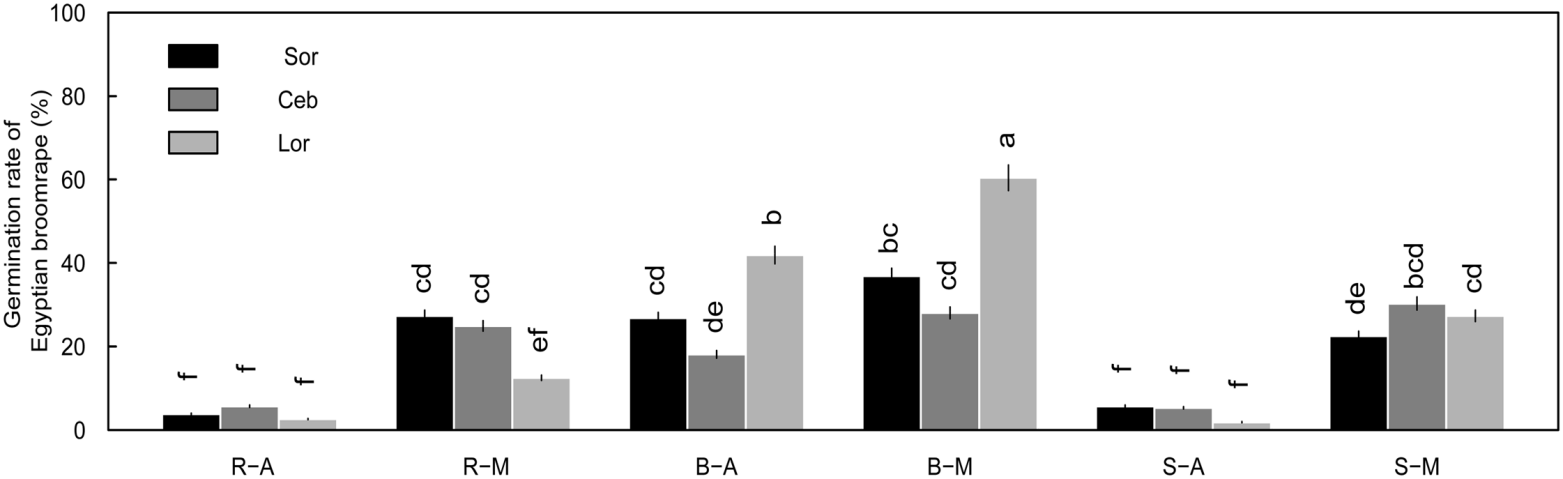
Germination of *O*. *aegyptiaca* Pers. induced by extracts of lily underground organs diluted 10-fold at the sprouting stage. Three cultivars of lily were assessed, Sorbonne, Ceb Dazzle and *L*. *formolongo*. Abbreviations: R-A, root aqueous extracts; R-M, root methanol extracts; B-A, bulb aqueous extracts; B-M, bulb methanol extracts; S-A, scale leaf aqueous extracts; S-M, scale leaf methanol extracts. Error bars represent the standard error of the mean. Different small letters above the error bars indicate significant differences at 0.05 (ANOVA and Duncan's multiple range test). Matching letters in the same color column indicates that there were no significant differences between treatments.

Of the underground organs, germination rates induced by the root and scale leaf were less than 9% when treated with aqueous extract (Fig 1A and 1C) (S1 Table), while germination rates from the aqueous and methanol extracts of bulbs were more than 20% in 10-fold and 100-fold dilutions, which exhibited relatively stable allelopathic effects on *O*. *aegyptiaca* Pers. seeds (Fig 1B) (S1 Table). Germination rates induced by 10-fold dilution of bulb extracts among the three lily cultivars were as follows: *L*. *formolongo* (41.9%) > Sorbonne (26.8%) > Ceb Dazzle (18.1%) in aqueous extracts and *L*. *formolongo* (60.4%) > Sorbonne (36.9%) > Ceb Dazzle (28.1%) in methanol extracts (Fig 2) (S1 Table). The differences among cultivars were significant (P<0.05), with *L*. *formolongo* bulbs inducing a significantly higher germination rate than the other two lily cultivars, whose allelopathic effects were similar.

### Induction of *O*. *aegyptiaca* Pers. Seed Germination at the Leaf Expansion Stage

At the leaf expansion stage, bulblets arose from the bulb base between two scales in which stored nutrients were being consumed. Lily above-ground organs grew vigorously and the flower bud was forming. The underground and above-ground organs were collected to test at stem height about 40–45 cm. Germination rates induced by methanol extracts for above- and underground organs were higher than aqueous extracts (S2 Table).

**Fig 3:**
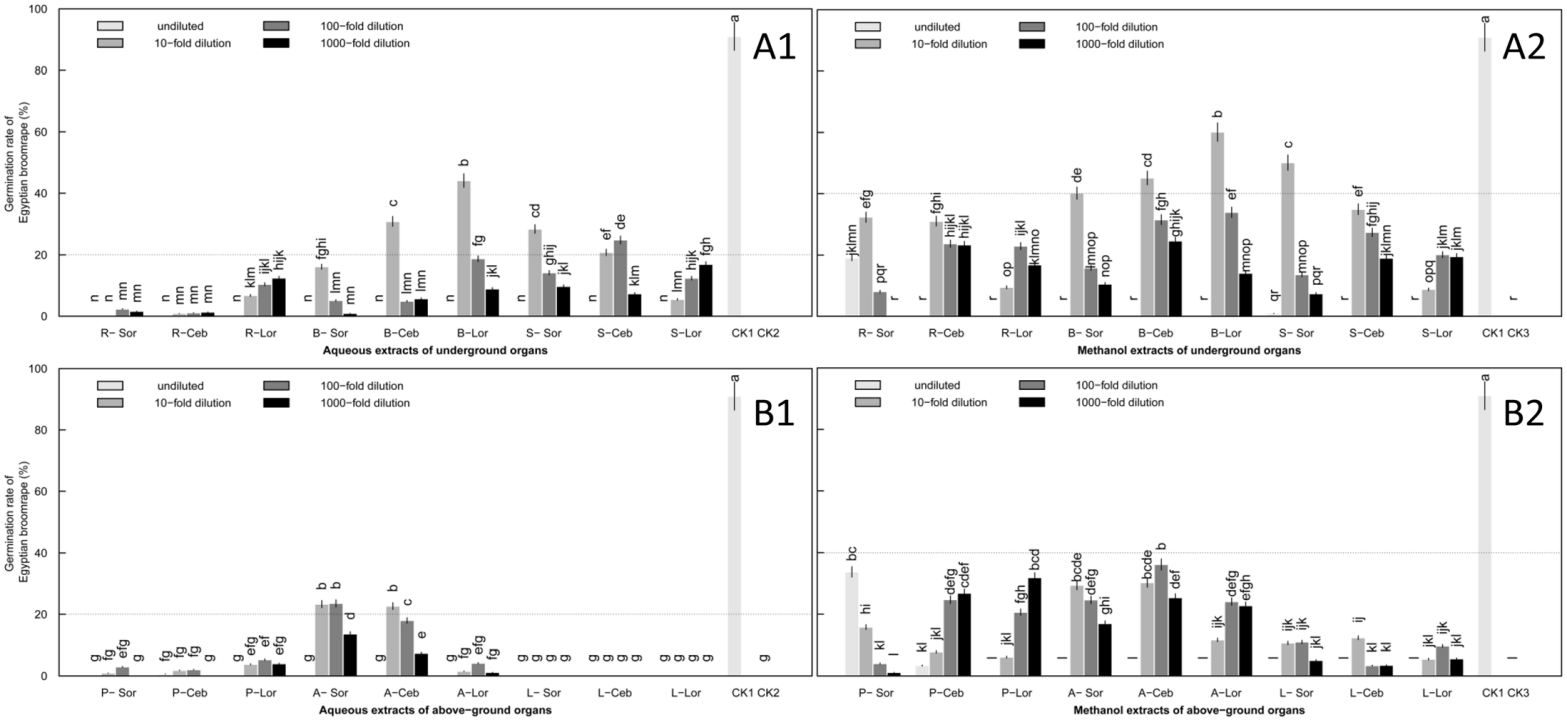
Induction of *O*. *aegyptiaca* Pers. germination by aqueous and methanol extracts of lily underground (A) and above-ground (B) organs at the leaf expansion stage. Two types of organs were tested, (A) underground organs and (B) above-ground organs. Two types of extracts were tested, (A1 and B1) aqueous and (A2 and B2) methanol. Four concentrations of extracts were assessed: undiluted and 10-fold, 100-fold and 1000-fold dilutions. Abbreviations: R-Sor, root extracts of Sorbonne; R-Ceb, root extracts of Ceb Dazzle; R-Lor, root extracts of *L*. formolongo; B-Sor, bulb extracts of Sorbonne; B-Ceb, bulb extracts of Ceb Dazzle; B-Lor, bulb extracts of *L*. formolongo; S-Sor, scale leaf extracts of Sorbonne; S-Ceb, scale leaf extracts of Ceb Dazzle; S-Lor, scale leaf extracts of *L*. formolongo; P-Sor, prop root extracts of Sorbonne; P-Ceb, prop root extracts of Ceb Dazzle; P-Lor, prop root extracts of *L*.*formolongo*; A-Sor, aerial stem extracts of Sorbonne; A-Ceb, aerial stem extracts of Ceb Dazzle; A-Lor, aerial stem extracts of *L*.*formolongo*.; L-Sor, leaf extracts of Sorbonne; L-Ceb, leaf extracts of Ceb Dazzle; L-Lor, leaf extracts of *L*.*formolongo*; CK1, GR24 as positive control; CK2, Distilled water as negative control; CK3, Methanol as negative control. The dotted black lines represent 20% and 40% position, respectively. Error bars represent the standard error of the mean. Different small letters above the error bars indicate significant differences at 0.05 (ANOVA and Duncan's multiple range test). Matching letters in the same color column indicates that there were no significant differences between treatments.

**Fig 4:**
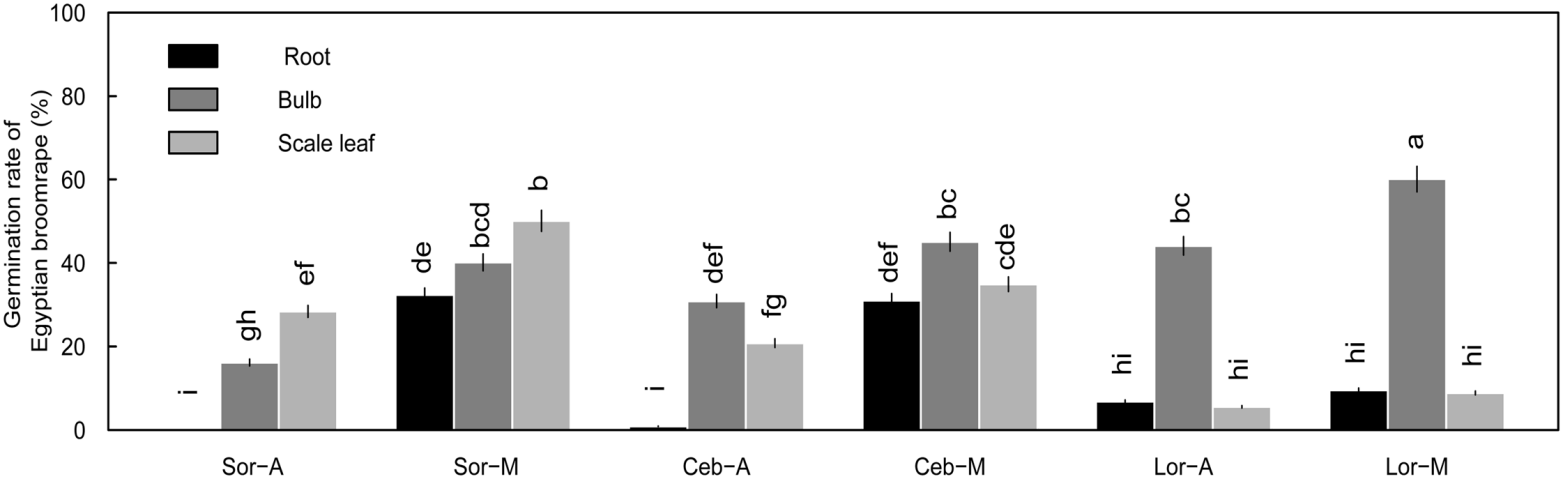
Germination of *O*. *aegyptiaca* Pers. induced by extracts of three lily cultivars diluted 10-fold at the leaf expansion stage. Lily underground organs were tested, including root, bulb and scale leaf. Abbreviations: Sor-A, Sorbonne aqueous extracts; Sor-M, Sorbonne methanol extracts; Ceb-A, Ceb Dazzle aqueous extracts; Ceb-M, Ceb Dazzle methanol extracts; Lor-A, *L*.*formolongo* aqueous extracts; Lor-M, *L*.*formolongo* methanol extracts. Error bars represent the standard error of the mean. Different small letters above the error bars indicate significant differences at 0.05 (ANOVA and Duncan's multiple range test). Matching letters in the same color column indicates that there were no significant differences between treatments.

The highest germination rates were induced by 10-fold dilution of aqueous and methanol extracts from underground organs (Fig 3). Maximum germination rates induced by 10-fold dilution of both aqueous and methanol extracts by organ were as follows: bulb > scale leaf > root in both Ceb Dazzle and *L*. *formolongo*. Differences between organs was significant (P<0.05) (Fig 4) (S2 Table). Ceb Dazzle (45.1%) and *L*. *formolongo* (60.1%) bulbs had relatively stable, strong allelopathic effects on *O*. *aegyptiaca* Pers. seeds, which were similar to effects in the sprouting stage (Figs 1 and 4). Although there were no significant differences between the underground organs of the Sorbonne cultivar, the highest germination rate was induced by scale leaf (50.1%), which was increased beyond the rate induced by bulbs in the leaf expansion stage (Fig 3A2).

Of the aqueous extracts from above-ground organs of the three lily cultivars, the highest germination rates induced by prop root and leaf were both less than 6% (Fig 3B1) (S2 Table). Allelopathy of Ceb Dazzle and Sorbonne aerial stems, which induced a maximum germination rate at or near 20%, was significantly stronger than that of *L*. *formolongo* (P<0.05) (Fig 3B1). For methanol extracts of above-ground organs, the highest germination rates induced by leaf extracts of the three lily cultivars were not more than 10% (S2 Table) and were significantly lower than rates induced by aerial stem or prop root extracts, which had germination rates of approximately 40%. Allelopathy was similar between aerial stem and prop root (Fig 3B2).

### Induction of *O*. *aegyptiaca* Pers. Seed Germination at the Flowering Stage

At the flowering stage, most of flower buds were blooming, which indicated that the lily was entering a reproductive phase. In particular, the smaller bulb of *L*. *formolongo* was completely consumed and its root declined at the flowering stage. Therefore, we were unable to test the allelopathy of underground organs of *L*. *formolongo* due to their disappearance at the flowering stage.

**Fig 5:**
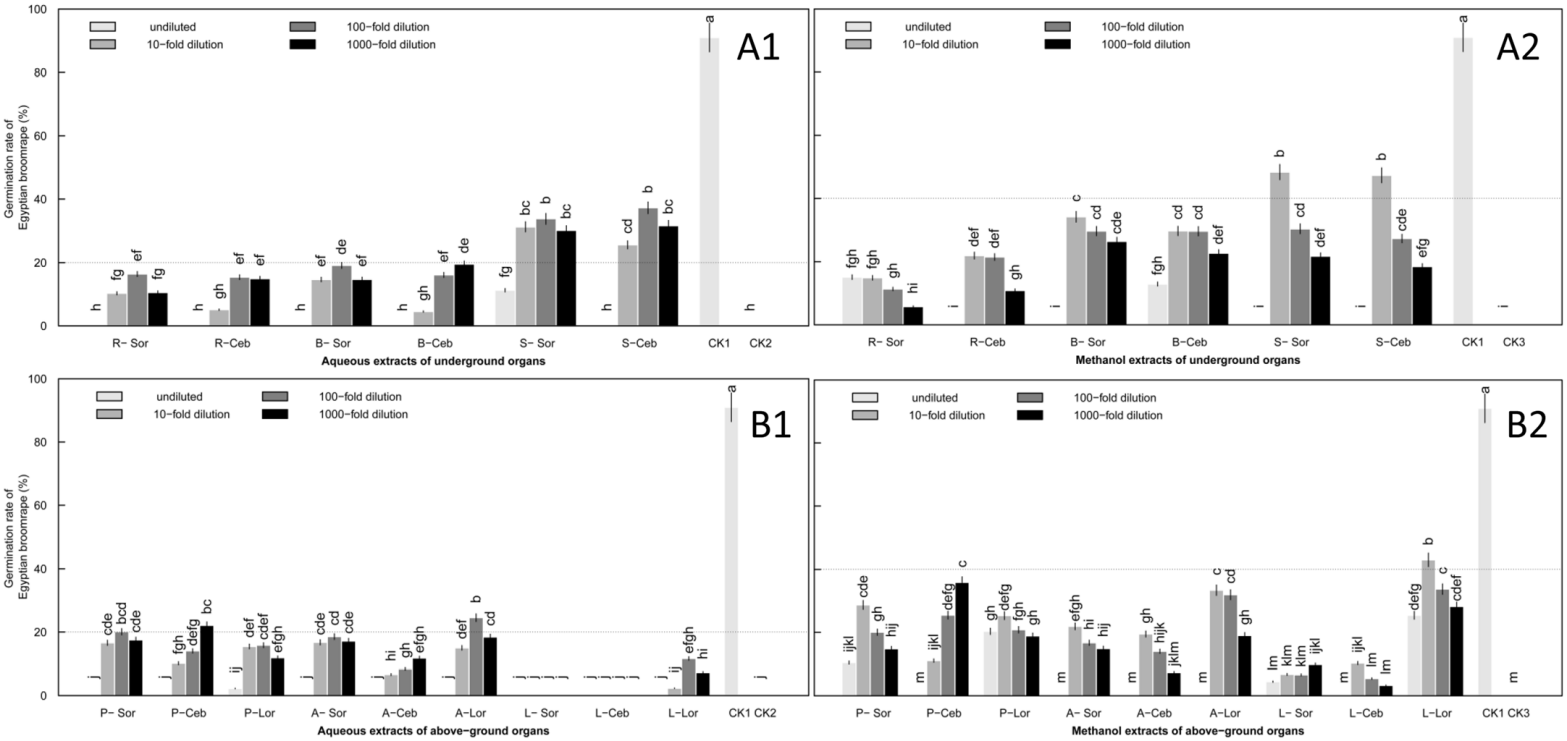
Induction of *O*. *aegyptiaca* Pers. germination by aqueous and methanol extracts of lily underground (A) and above-ground (B) organs at the flowering stage. Two types of organs were tested, (A) underground organs and (B) above-ground organs. Two types of extracts were tested, (A1 and B1) aqueous and (A2 and B2) methanol. Four concentrations of extracts were assessed: undiluted and 10-fold, 100-fold and 1000-fold dilutions. Abbreviations: R-Sor, root extracts of Sorbonne; R-Ceb, root extracts of Ceb Dazzle; B-Sor, bulb extracts of Sorbonne; B-Ceb, bulb extracts of Ceb Dazzle; S-Sor, scale leaf extracts of Sorbonne; S-Ceb, scale leaf extracts of Ceb Dazzle; P-Sor, prop root extracts of Sorbonne; P-Ceb, prop root extracts of Ceb Dazzle; P-Lor, prop root extracts of *L*.*formolongo*.; A-Sor, aerial stem extracts of Sorbonne; A-Ceb, aerial stem extracts of Ceb Dazzle; A-Lor, aerial stem extracts of *L*.*formolongo*.; L-Sor, leaf extracts of Sorbonne; L-Ceb, leaf extracts of Ceb Dazzle; L-Lor, leaf extracts of *L*.*formolongo*; CK1, GR24 as positive control; CK2, Distilled water as negative control; CK3, Methanol as negative control. The dotted black lines represent 20% and 40% position, respectively. Error bars represent the standard error of the mean. Different small letters above the error bars indicate significant differences at 0.05 (ANOVA and Duncan's multiple range test). Matching letters in the same color column indicates that there were no significant differences between treatments.

Whether extracted by water or methanol, underground scale leaf from both lily cultivars tested possessed stronger allelopathic stimulants compared to other organs. Specifically, when methanol extracts were diluted 10-fold, the order of allelopathic effects among the organs was scale leaf > bulb > root. Maximum germination rates induced by scale leaf of both lily cultivars were more than 40%, which was significantly better than rates induced by the other two organs (P<0.05) (Fig 5A1). When scale leaf aqueous extracts were diluted 100-fold, allelopathy in both lily cultivars that induced the highest germination rates were close to 40%. This was significantly higher than germination rates induced by root and bulb, which were close to 20% (P<0.05) (Fig 5A1). Maximum germination rates induced by each organ from Ceb Dazzle were very similar to rates induced by the same organ from Sorbonne (S3 Table). There were no significant differences in allelopathy between Ceb Dazzle and Sorbonne (P<0.05) (Fig 5).

For above-organs, *L*. *formolongo* allelopathy was apparently different from the other two lily cultivars. The highest germination rates induced by prop root and aerial stem aqueous extracts of the three lily cultivars were near 20%, followed by 10% which was induced by leaves (Fig 5B1) (S3 Table). For prop root, there were no significant differences among the lily cultivars, while the other two organs from *L*. *formolongo* induced significantly higher germination rates than Sorbonne and Ceb Dazzle (P<0.05) (Fig 5B1). Methanol extracts of aerial stem and leaves showed identical variation tendencies as aqueous extracts, while results for prop root from *L*. *formolongo* on contrary, which induced significantly lower germination rates compared to other organs (P<0.05) (Fig 5B2).

### Induction of *O*. *aegyptiaca* Pers. Seed Germination at the Increasing Bulblet Weight Stage

At the increasing bulblet weight stage, nutrients generated by above-ground parts are transported to the underground organs to support growth of the next bulblet after the flowers withered.

**Fig 6:**
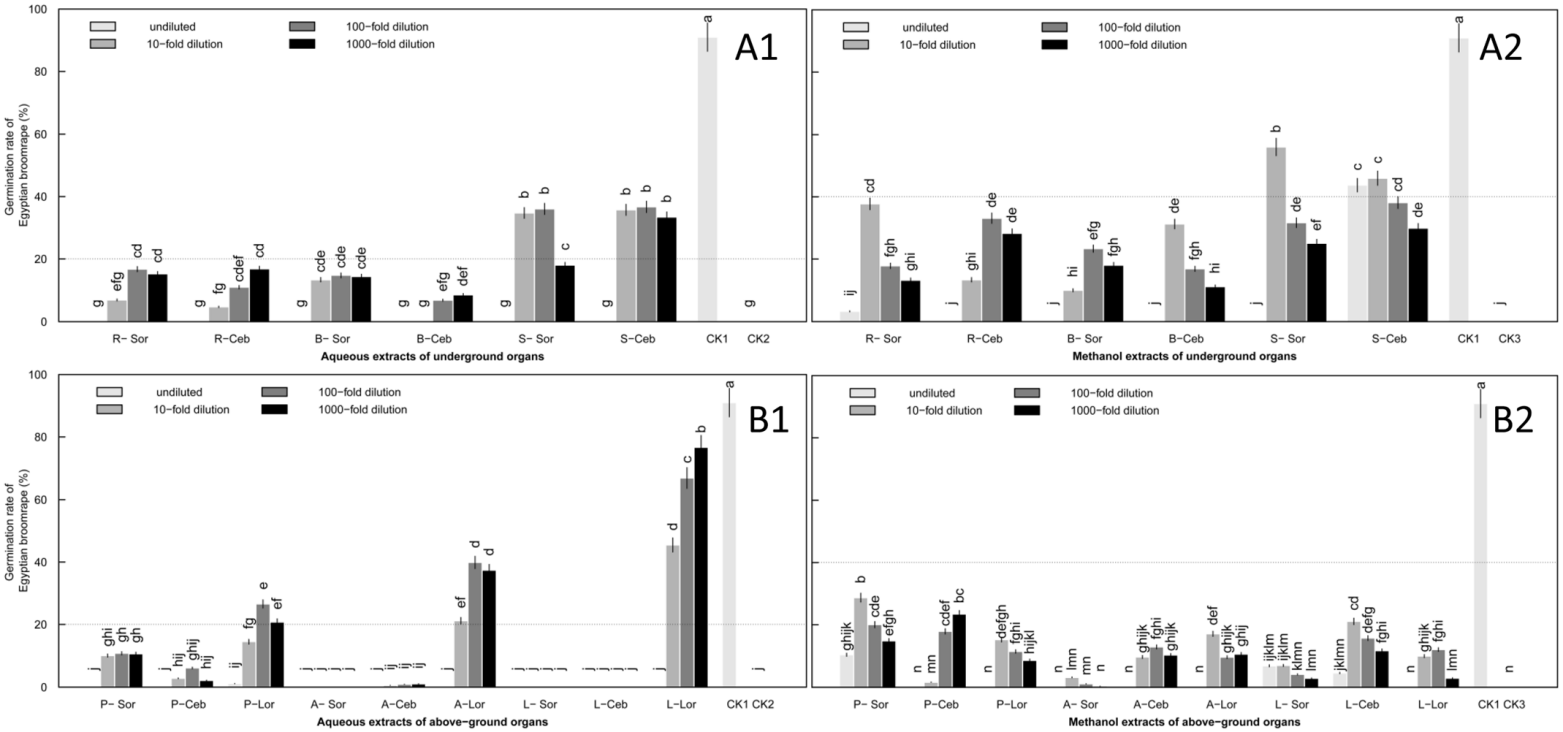
Induction of *O*. *aegyptiaca* Pers. germination by aqueous and methanol extracts of lily underground (A) and above-ground (B) organs at the increasing bulblet weight stage. Two types of organs were tested, (A) underground organs and (B) above-ground organs. Two types of extracts were tested, (A1 and B1) aqueous and (A2 and B2) methanol. Four concentrations of extracts were assessed: undiluted and 10-fold, 100-fold and 1000-fold dilutions. Abbreviations: R-Sor, root extracts of Sorbonne; R-Ceb, root extracts of Ceb Dazzle; B-Sor, bulb extracts of Sorbonne; B-Ceb, bulb extracts of Ceb Dazzle; S-Sor, scale leaf extracts of Sorbonne; S-Ceb, scale leaf extracts of Ceb Dazzle; P-Sor, prop root extracts of Sorbonne; P-Ceb, prop root extracts of Ceb Dazzle; P-Lor, prop root extracts of *L*.*formolongo*; A-Sor, aerial stem extracts of Sorbonne; A-Ceb, aerial stem extracts of Ceb Dazzle; A-Lor, aerial stem extracts of *L*.*formolongo*; L-Sor, leaf extracts of Sorbonne; L-Ceb, leaf extracts of Ceb Dazzle; L-Lor, leaf extracts of *L*.*formolongo*; CK1, GR24 as positive control; CK2, Distilled water as negative control; CK3, Methanol as negative control. The dotted black lines represent 20% and 40% position, respectively. The Error bars represent the standard error of the mean. Error bars represent the standard error of the mean. Different small letters above the error bars indicate significant differences at 0.05 (ANOVA and Duncan's multiple range test). Matching letters in the same color column indicates that there were no significant differences between treatments.

The scale leaf was the strongest allelopathic stimulant. There was a significant difference in inducement of *O*. *aegyptiaca* Pers. germination between lily cultivars in the scale leaf development period. In comparing cultivars, the scale leaf extracts from Ceb Dazzle and Sorbonne exhibited similar maximum germination rates relative to other developmental stages, with 40% and 60%, respectively, in aqueous and methanol extracts (Fig 6A). Inducement of *O*. *aegyptiaca* Pers. seed germination was lower in the root and bulb, with only 20% and 40% in aqueous and methanol extracts, respectively.

For aqueous extracts of above-ground organs, it was notable that *L*. *formolongo* leaves induced the highest germination rate (76.7%), with allelopathic effects that were similar to the synthetic strigolactone GR24 (Fig 6B1). Furthermore, allelopathy of all *L*. *formolongo* above-ground organs was significantly stronger than Ceb Dazzle and Sorbonne cultivars, which had germination rates less than 11% from aqueous extracts (P<0.05) (S4 Table). Unlike aqueous extracts, Ceb Dazzle and Sorbonne prop root methanol extracts induced significantly higher germination rates than *L*. *formolongo* (P<0.05). The highest germination rates induced by the Sorbonne aerial stems and leaves were less than 8% (S4 Table), which was significantly lower than that of the other two lily cultivars (P<0.05) (Fig 6B2). Additionally, Ceb Dazzle and Sorbonne methanol extracts induced higher germination rates than aqueous extracts, but *L*. *formolongo* had an opposite result (Fig 6B).

### Induction of *O*. *aegyptiaca* Pers. Seed Germination by Rhizosphere Soil

**Fig 7:**
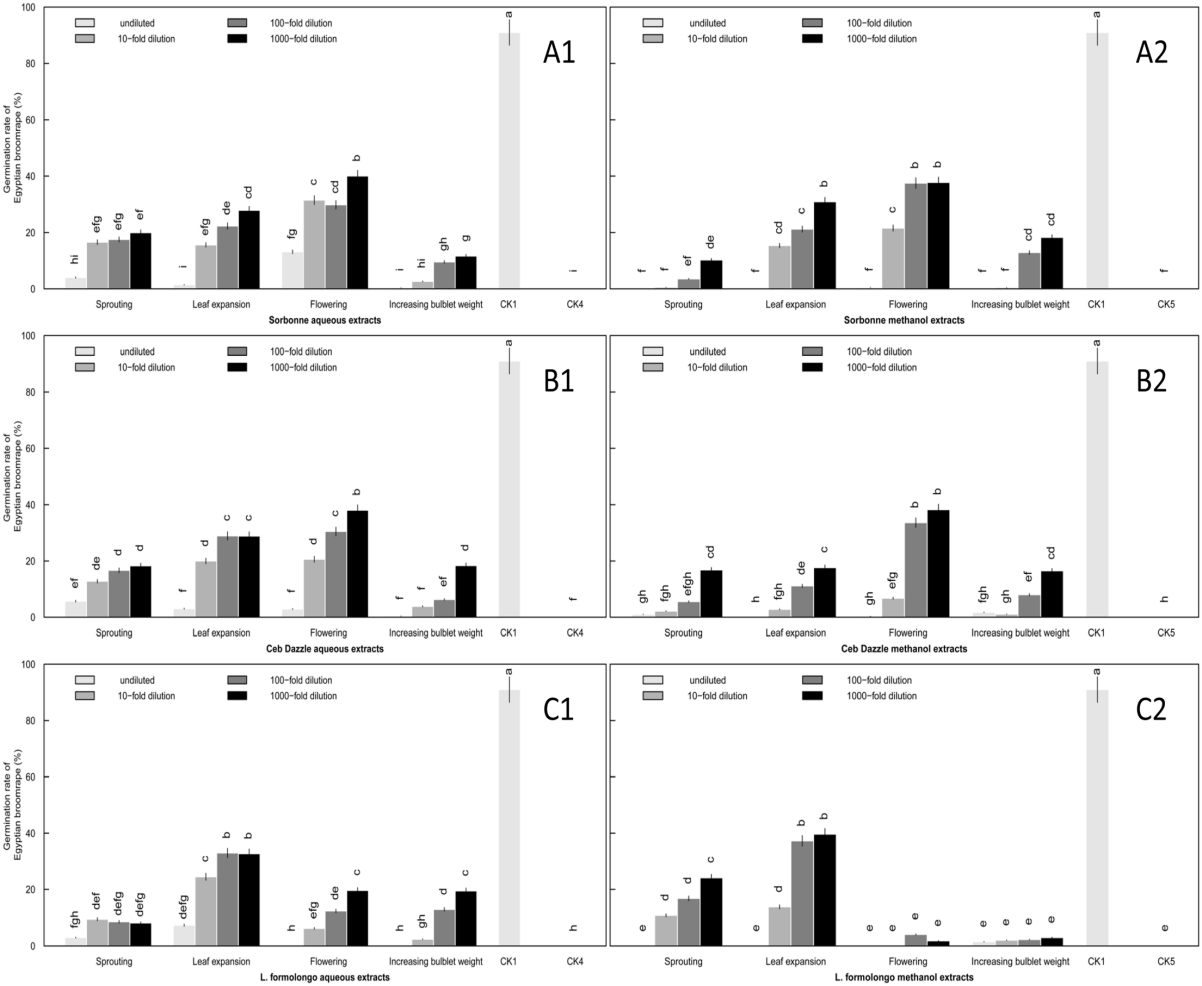
Induction of *O*. *aegyptiaca* Pers. germination by rhizosphere soil extracts of three lily cultivars Sorbonne (A), Ceb Dazzle (B) and L.*formolongo* (C) at the different growth stage. Three cultivars of lily were assessed (A) Sorbonne, (B) Ceb Dazzle and (C) *L*.*formolongo*. Two types of extracts were tested, (A1, B1 and C1) aqueous and (A2, B2 and C2) methanol. Four concentrations of extracts were assessed: undiluted and 10-fold, 100-fold and 1000-fold dilutions. Abbreviations: CK1, GR24 as positive control; CK4, Aqueous extract of cultivation medium as negative control; CK5, Methanol extract of cultivation medium as negative control. Error bars represent the standard error of the mean. Different small letters above the error bars indicate significant differences at 0.05 (ANOVA and Duncan's multiple range test). Matching letters in the same color column indicates that there were no significant differences between treatments.

Rhizosphere soil from the three lily cultivars induced *O*. *aegyptiaca* Pers. seed germination at all four growth stages. Germination rates induced by aqueous extracts were higher or similar to methanol extracts (S5 Table). Few *O*. *aegyptiaca* Pers. seeds were germinated with undiluted aqueous and methanol extracts. Germination rates gradually increased with aqueous and methanol extract dilutions, and the highest germination rates were induced by extracts diluted 1000-fold (Fig 7). The majority of differences in germination rates between Sorbonne and Ceb Dazzle cultivars under the same dilution concentrations were less than between them and *L*. *formolongo*.

**Fig 8:**
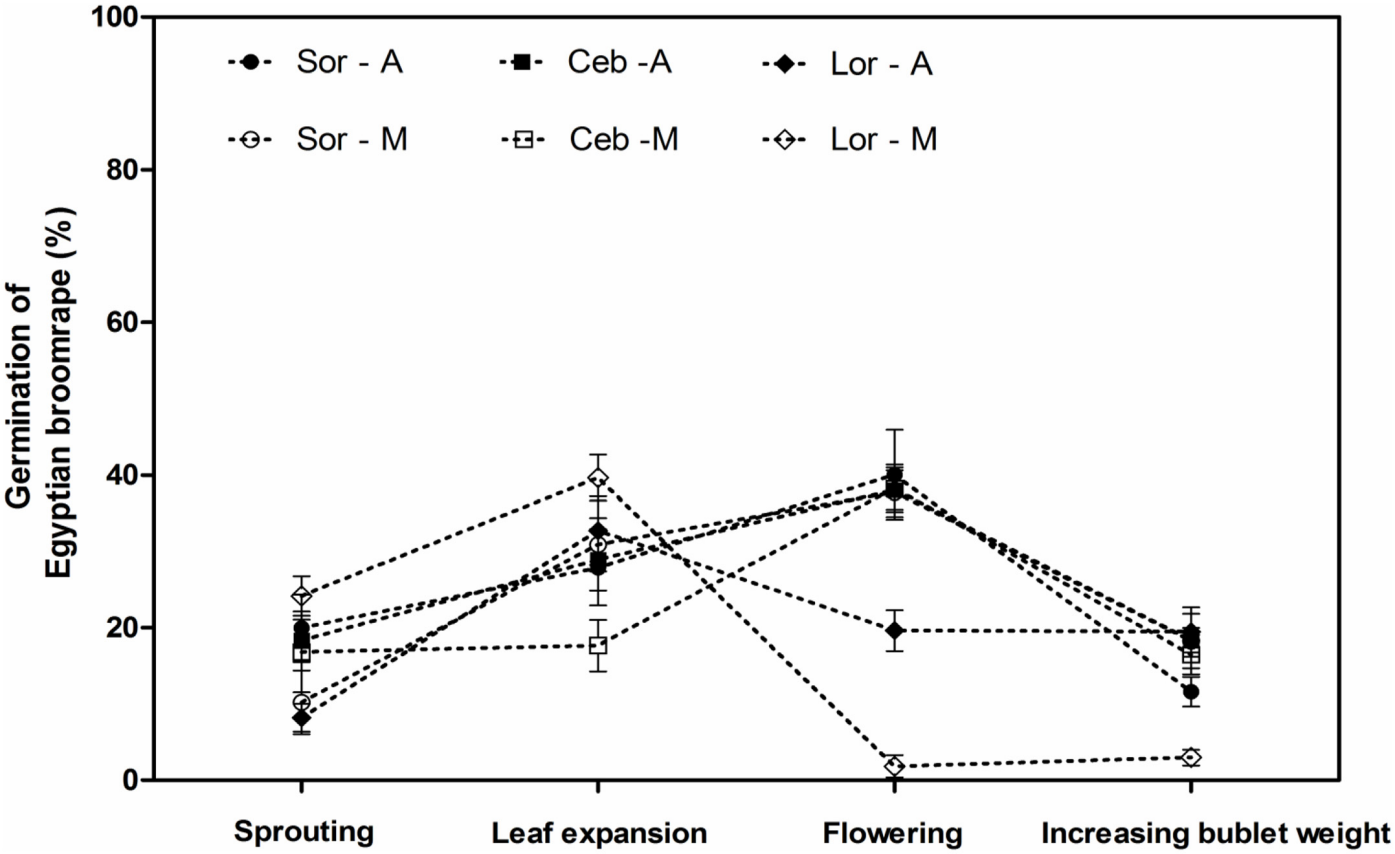
Germination of *O*. *aegyptiaca* Pers. induced by extracts of lily rhizosphere soil diluted 1000-fold at the different growth stages. Abbreviations: Sor-A, Sorbonne aqueous extracts; Sor-M, Sorbonne methanol extracts; Ceb–A, Ceb Dazzle aqueous extracts; Ceb–M, Ceb Dazzle methanol extracts; Lor-A, *L*.*formolongo* aqueous extracts; Lor-M, *L*.*formolongo* methanol extracts. Error bars represent the standard error of the mean. Different small letters above the error bars indicate significant differences at 0.05 (ANOVA and Duncan's multiple range test). Matching letters in the same color column indicates that there were no significant differences between treatments.

By analyzing variation in germination rates induced by aqueous and methanol extracts diluted 1000-fold at various stages of lily growth, it is found that the curve variation tendencies of Ceb Dazzle and Sorbonne were approximate, the highest germination rates appeared in the flowering stage, while the strongest stimulant allelopathy of *L*. *formolongo* appeared in leaf expansion stage and then the germination rates decreased rapidly (Fig 8).

## Discussion

### Rhizosphere Soil from Lily Cultivars Stimulates *O*. *aegyptiaca* Pers. Seed Germination

Rhizosphere soil extracts from three different lily cultivars induced parasitic *O*. *aegyptiaca* Pers. seed germination at all four growth stages, showing the strongest allelopathic effects at the flowering stage (Fig 7). This suggests that lily roots may continuously secrete allelopathic compounds into the soil over time.

Germination rates induced by lily rhizosphere soil extracts varied significantly depending on concentration (Fig 7). Researchers have noted that most allelopathic substances stimulate seed germination at low concentrations and inhibit germination at high concentrations [23, 36, 37], which is in agreement with the results of the present study. Specifically, undiluted rhizosphere soil aqueous and methanol extracts induced little or no germination of *O*. *aegyptiaca* Pers., while most inhibitory effects vanished and germination rates gradually increased when extracts were diluted to 1000-fold. This indicates that *O*. *aegyptiaca* Pers. seed germination could be stimulated within a considerable distance of lily roots. Future studies should investigate whether germination rates continue to increase under more dilute conditions.

Some researchers have reported that methanol extracts generally induce higher germination rates than aqueous extracts. One possible explanation was that extracts might contain unknown germination inhibitors that might not remain stable and could become volatile in the presence of methanol [38, 39]. However, the results of the present study were not consistent with a previous study in which germination rates induced by aqueous lily rhizosphere soil extracts were higher or similar to methanol extracts (Fig 7). On the one hand, we speculated that aqueous lily rhizosphere soil extracts might contain more hydrophilic allelochemicals or fewer unknown inhibitors, suggesting that stimulant allelopathic effects from lily rhizosphere soil on *O*. *aegyptiaca* Pers. seeds would be stronger under leaching conditions [20, 40]. One the other hand, some researchers found that environmental conditions affected production and/or exudation of SLs known as the major germination stimulants of *Striga* and *Orobanche*. For example, it have already been proved that SLs exudation is promoted in response to soil nutrient deficiency and the stability of SLs rapidly would decrease at alkaline condition [31, 33, 41]. Therefore, taking these factors into consideration, we also speculated that slightly acid soil condition of lily might protect these compounds from disruption and decomposition.

In addition, the strongest stimulating allelopathic effects of rhizosphere soil extracts from Sorbonne and Ceb Dazzle were exhibited at the flowering stage, while the effects were shown at the leaf expansion stage for *L*. *formolongo* (Fig 8). Similarly, the study of the root exudates of tomato (*Lycopersicon esculentum* Mill.) genotypes effect on seed germination of *Orobanche* has revealed that significant variations of germination-stimulating capacities in relation to plant age, probably because of different production of stimulant [42]. It also have been confirmed that root exudates of peanut (*Arachis hypogaea L*.) exhibit qualitative and quantitative changes in their composition with the increasing age of the plant [43]. Therefore, it could be that type and concentration of allelopathic substances might vary at the various growth stage of lily, which caused that allelopathy varies based on lily growth and *L*. *formolongo* has different allelopathic effects compared to the other two cultivars. Overall, these results suggest that lily cultivars have the potential to be used as trap crops to reduce *O*. *aegyptiaca* Pers. seed banks.

### Allelopathic Effects on *O*. *aegyptiaca* Pers. Seed Germination may Vary among Lily Cultivars or Organs

It has been reported that one single plant species can produce several different types and quantities of strigolactones, and within the same species, different varieties may also produce different combinations of strigolactones [19, 42, 45]. This conclusion is similar to findings in grain crop and vegetable crop studies, such as soybean, wheat, cotton and hybrid, in which allelopathy can vary dramatically among cultivars [10–14, 44, 46]. Comparing allelopathy among above- and underground organs from three different lily cultivars, it was determined that *L*. *formolongo* was significantly different in the ability to stimulate *O*. *aegyptiaca* Pers. seed germination with respect to the Sorbonne and Ceb Dazzle cultivars, which had similar allelopathic effects.

**Fig 9:**
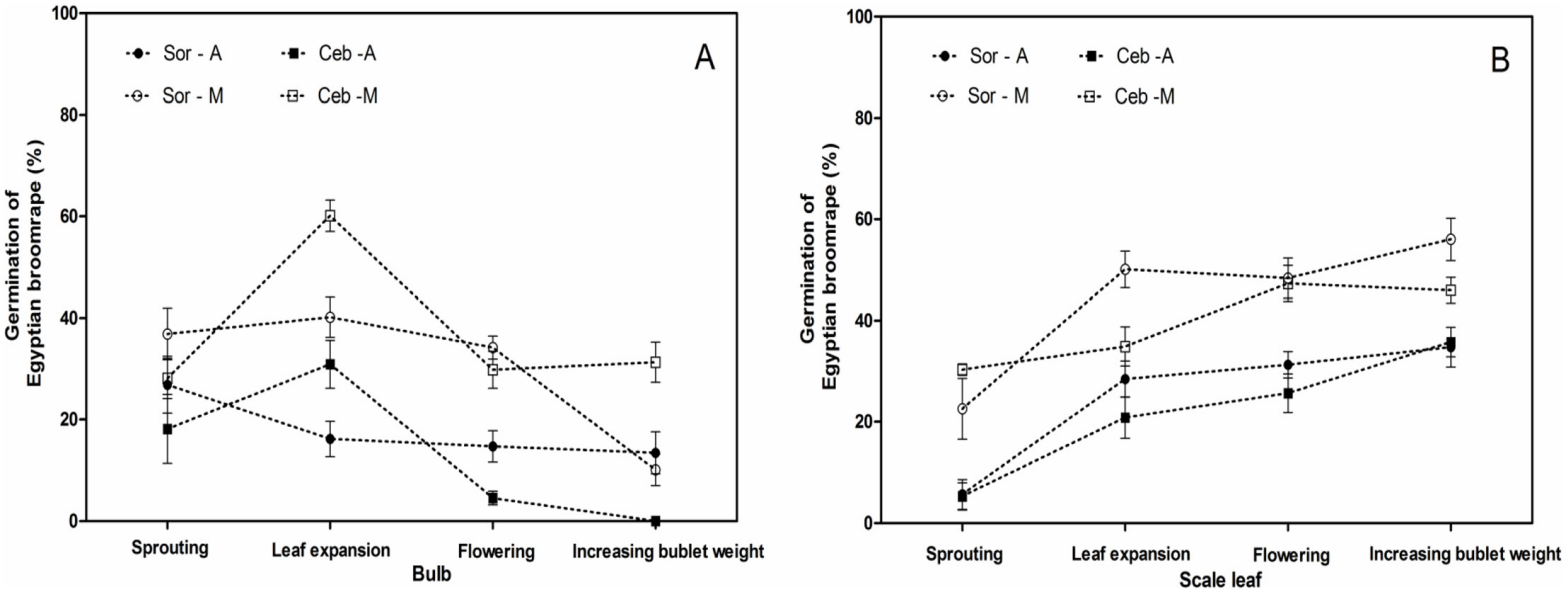
Germination of *O*. *aegyptiaca* Pers. induced by lily bulb (A) and scale leaf (B) extracts diluted 10-fold at different growth stages. (A) Bulb and (B) scale leaf organs were compared. Abbreviations: Sor-A, Sorbonne aqueous extracts; Sor-M, Sorbonne methanol extracts; Ceb–A, Ceb Dazzle aqueous extracts; Ceb–M, Ceb Dazzle methanol extracts. Error bars represent the standard error of the mean. Different small letters above the error bars indicate significant differences at 0.05 (ANOVA and Duncan's multiple range test). Matching letters in the same color column indicates that there were no significant differences between treatments.

For Sorbonne and Ceb Dazzle, bulb and scale leaf from the underground organs exhibited the strongest stimulant allelopathy, and above-ground organs from both cultivars generally had weaker stimulant allelopathy at the same growth stage. Regardless of growth stage, aqueous and methanol leaf extracts had the lowest capacity to induce *O*. *aegyptiaca* Pers. seed germination (Figs 3B, 5B and 6B). However, these results were not consistent with recent studies in which allelopathic substances produced by cotton and potato root were transported upwards and retained in above-ground parts, resulting in a positive relationship between root and stem or leaf [13, 20, 47]. Instead, our results showed that a transition between organ types occurred at a specific turning point (i.e., leaf expansion stage), with the strongest stimulant allelopathy from the bulb to scale leaf in underground organs during lily growth (Fig 9). Actually, recent experiments have demonstrated that the function of SLs or their metabolites in the rhizosphere were synthesized in both roots and stem and transported upward in xylem for control of shoot branching. This means that SLs may have the certain mode of action and exist in some parts of plant [13, 20, 47]. Based on these facts, we presume that sophisticated components of the bulbous lily plant are likely the main causes of a distinct allelochemical transportation model, which should be further investigated.

At the first two growth stages in *L*. *formolongo*, underground bulbs had the strongest allelopathic effect (Figs 1 and 3A), followed by above-ground leaves at the last two growth stages (Figs 5B and 6B). More importantly, germination rates induced by aqueous leaf extracts at the increasing bulblet weight stage were significantly improved and reached a maximum rate of 76.7% with increased extract dilutions, which had a similar effect as the synthetic strigolactone analogue GR24 [40, 48]. In facts, SLs inducing germination of parastic weeds have been found that contain hydroxy-SLs, such as strigol, orobanchol and sorgomol than non-hydroxy-SLs such as sorgolactone and 5-deoxystrigol [40]. Therefore, it was hypothesized that *L*. *formolongo* leaves may contain more stable water soluble stimulants, having the potential to be processed into green herbicide formulations with wide applications in agriculture production to rapidly deplete the seed bank of *O*. *aegyptiaca* Pers. parasitic weeds in soil.

## Supporting Information

S1 Table *O*. *Aegyptiaca* Pers. seed germination induced by aqueous and methanol extracts of underground organs from three lily cultivars at the sprouting stage. (DOCX)

S2 Table *O*. *Aegyptiaca* Pers. seed germination induced by aqueous and methanol extracts of above- and underground organs from three lily cultivars at the leaf expansion stage. (DOCX)

S3 Table *O*. *Aegyptiaca* Pers. seed germination induced by aqueous and methanol extracts of above- and underground organs from three lily cultivars at the flowering stage. (DOCX)

S4 Table *O*. *Aegyptiaca* Pers. seed germination induced by aqueous and methanol extracts of above- and underground organs from three lily cultivars at the increasing bulblet weight stage. (DOCX)

S5 Table *O*. *Aegyptiaca* Pers. seed germination induced by aqueous and methanol extracts of rhizosphere soil from three lily cultivars at different growth stages. (DOCX)
