# Supplementary material for: Lily Cultivars Have Allelopathic Potential in Controlling Orobanche aegyptiaca Persoon
Source: PLoS One. 2015 Nov 13;10(11):e0142811. doi: 10.1371/journal.pone.0142811 (PMC4643976; doi:10.1371/journal.pone.0142811)
Supplement: S1 Table — (DOCX) [file pone.0142811.s001.docx]

**S1A Table O. aegyptiaca seeds germination induced by aqueous and methanol extracts of three cultivars lily below-organs (Root) at sprouting growth stage.**

| Below-organs (root) | | | | | | | | | |
| --- | --- | --- | --- | --- | --- | --- | --- | --- | --- |
| N | Sample | Concentration | Difference | Mean | N | Sample | Concentration | Difference | Mean |
| 1 | Sor-A | undiluted | h | 0 | 13 | Ceb-M | undiluted | h | 0 |
| 2 | Sor-A | 10-fold dilution | fgh | 3.77068 | 14 | Ceb-M | 10-fold dilution | b | 24.95030 |
| 3 | Sor-A | 100-fold dilution | fgh | 5.05734 | 15 | Ceb-M | 100-fold dilution | b | 24.89787 |
| 4 | Sor-A | 1000-fold dilution | efgh | 6.28934 | 16 | Ceb-M | 1000-fold dilution | bc | 22.00262 |
| 5 | Sor-M | undiluted | h | 0 | 17 | Lor-A | undiluted | h | 0 |
| 6 | Sor-M | 10-fold dilution | b | 27.35207 | 18 | Lor-A | 10-fold dilution | gh | 2.55540 |
| 7 | Sor-M | 100-fold dilution | b | 22.93158 | 19 | Lor-A | 100-fold dilution | gh | 1.85507 |
| 8 | Sor-M | 1000-fold dilution | cd | 16.46073 | 20 | Lor-A | 1000-fold dilution | h | 0 |
| 9 | Ceb-A | undiluted | h | 0 | 21 | Lor-M | undiluted | h | 0 |
| 10 | Ceb-A | 10-fold dilution | efgh | 6.12587 | 22 | Lor-M | 10-fold dilution | de | 12.47355 |
| 11 | Ceb-A | 100-fold dilution | ef | 9.72833 | 23 | Lor-M | 100-fold dilution | efg | 8.10707 |
| 12 | Ceb-A | 1000-fold dilution | efgh | 6.84975 | 24 | Lor-M | 1000-fold dilution | efgh | 5.78814 |

Abbreviations: Sor-A, Sorbone aqueous extracts; Sor-M, Sorbone methanol extracts; Ceb–A, Ceb Dazzle aqueous extracts; Ceb–M, Ceb Dazzle methanol extracts; Lor-A, *L.formolongo* aqueous extracts; Lor-M, *L.formolongo* methanol extracts.

**S1B Table O. aegyptiaca seeds germination induced by aqueous and methanol extracts of three cultivars lily below-organs (Bulb) at sprouting growth stage.**

| Below-organs (bulb) | | | | | | | | | |
| --- | --- | --- | --- | --- | --- | --- | --- | --- | --- |
| N | Sample | Concentration | Difference | Mean | N | Sample | Concentration | Difference | Mean |
| 1 | Sor-A | undiluted | h | 0 | 13 | Ceb-M | undiluted | h | 0 |
| 2 | Sor-A | 10-fold dilution | de | 26.84016 | 14 | Ceb-M | 10-fold dilution | de | 28.07931 |
| 3 | Sor-A | 100-fold dilution | ef | 18.91019 | 15 | Ceb-M | 100-fold dilution | cd | 36.15239 |
| 4 | Sor-A | 1000-fold dilution | ef | 17.64093 | 16 | Ceb-M | 1000-fold dilution | fg | 13.52018 |
| 5 | Sor-M | undiluted | h | 0 | 17 | Lor-A | undiluted | h | 0 |
| 6 | Sor-M | 10-fold dilution | cd | 36.86738 | 18 | Lor-A | 10-fold dilution | c | 41.90575 |
| 7 | Sor-M | 100-fold dilution | cd | 34.28351 | 19 | Lor-A | 100-fold dilution | fg | 14.90090 |
| 8 | Sor-M | 1000-fold dilution | fg | 14.69586 | 20 | Lor-A | 1000-fold dilution | h | 1.47368 |
| 9 | Ceb-A | undiluted | h | 0 | 21 | Lor-M | undiluted | h | 0 |
| 10 | Ceb-A | 10-fold dilution | ef | 18.11007 | 22 | Lor-M | 10-fold dilution | b | 60.41693 |
| 11 | Ceb-A | 100-fold dilution | ef | 17.57617 | 23 | Lor-M | 100-fold dilution | cd | 35.24944 |
| 12 | Ceb-A | 1000-fold dilution | gh | 5.32324 | 24 | Lor-M | 1000-fold dilution | ef | 21.06189 |

Abbreviations: Sor-A, Sorbone aqueous extracts; Sor-M, Sorbone methanol extracts; Ceb–A, Ceb Dazzle aqueous extracts; Ceb–M, Ceb Dazzle methanol extracts; Lor-A, *L.formolongo* aqueous extracts; Lor-M, *L.formolongo* methanol extracts.

**S1C Table O. aegyptiaca seeds germination induced by aqueous and methanol extracts of three cultivars lily below-organs (Scale leaf) at sprouting growth stage.**

| Below-organs (scale leaf) | | | | | | | | | |
| --- | --- | --- | --- | --- | --- | --- | --- | --- | --- |
| N | Sample | Concentration | Difference | Mean | N | Sample | Concentration | Difference | Mean |
| 1 | Sor-A | undiluted | j | 0 | 13 | Ceb-M | undiluted | ij | 1.36390 |
| 2 | Sor-A | 10-fold dilution | fghij | 5.63412 | 14 | Ceb-M | 10-fold dilution | b | 30.29724 |
| 3 | Sor-A | 100-fold dilution | fg | 8.48922 | 15 | Ceb-M | 100-fold dilution | de | 17.97363 |
| 4 | Sor-A | 1000-fold dilution | fghij | 4.48158 | 16 | Ceb-M | 1000-fold dilution | e | 15.62544 |
| 5 | Sor-M | undiluted | j | 0 | 17 | Lor-A | undiluted | j | 0 |
| 6 | Sor-M | 10-fold dilution | cd | 22.52716 | 18 | Lor-A | 10-fold dilution | hij | 1.85877 |
| 7 | Sor-M | 100-fold dilution | f | 9.84333 | 19 | Lor-A | 100-fold dilution | f | 9.36147 |
| 8 | Sor-M | 1000-fold dilution | ij | 1.46150 | 20 | Lor-A | 1000-fold dilution | fgh | 8.10464 |
| 9 | Ceb-A | undiluted | j | 0 | 21 | Lor-M | undiluted | j | 0 |
| 10 | Ceb-A | 10-fold dilution | fghij | 5.25371 | 22 | Lor-M | 10-fold dilution | bc | 27.36081 |
| 11 | Ceb-A | 100-fold dilution | fghi | 6.87697 | 23 | Lor-M | 100-fold dilution | b | 29.97663 |
| 12 | Ceb-A | 1000-fold dilution | ghij | 2.20891 | 24 | Lor-M | 1000-fold dilution | de | 18.39480 |

Abbreviations: Sor-A, Sorbone aqueous extracts; Sor-M, Sorbone methanol extracts; Ceb–A, Ceb Dazzle aqueous extracts; Ceb–M, Ceb Dazzle methanol extracts; Lor-A, *L.formolongo* aqueous extracts; Lor-M, *L.formolongo* methanol extracts.
