# Supplementary material for: Lily Cultivars Have Allelopathic Potential in Controlling Orobanche aegyptiaca Persoon
Source: PLoS One. 2015 Nov 13;10(11):e0142811. doi: 10.1371/journal.pone.0142811 (PMC4643976; doi:10.1371/journal.pone.0142811)
Supplement: S2 Table — (DOCX) [file pone.0142811.s002.docx]

**S2A Table *O. aegyptiaca* seeds germination induced by aqueous extracts of three cultivars lily below-organs at leaf-expanding growth stage.**

| Below-organs aqueous | | | | | | | | | |
| --- | --- | --- | --- | --- | --- | --- | --- | --- | --- |
| N | Sample | Concentration | Difference | Mean | N | Sample | Concentration | Difference | Mean |
| 1 | R-Sor | undiluted | n | 0 | 19 | B-Ceb | 100-fold dilution | lmn | 4.91369 |
| 2 | R-Sor | 10-fold dilution | n | 0 | 20 | B-Ceb | 1000-fold dilution | lmn | 5.71829 |
| 3 | R-Sor | 100-fold dilution | mn | 2.34303 | 21 | B-Lor | undiluted | n | 0 |
| 4 | R-Sor | 1000-fold dilution | mn | 1.61523 | 22 | B-Lor | 10-fold dilution | b | 44.10467 |
| 5 | R-Ceb | undiluted | n | 0 | 23 | B-Lor | 100-fold dilution | fg | 18.76239 |
| 6 | R-Ceb | 10-fold dilution | mn | 0.90278 | 24 | B-Lor | 1000-fold dilution | jkl | 8.92213 |
| 7 | R-Ceb | 100-fold dilution | mn | 1.10390 | 25 | S-Sor | undiluted | n | 0 |
| 8 | R-Ceb | 1000-fold dilution | mn | 1.33895 | 26 | S-Sor | 10-fold dilution | cd | 28.43279 |
| 9 | R-Lor | undiluted | n | 0 | 27 | S-Sor | 100-fold dilution | ghij | 14.17450 |
| 10 | R-Lor | 10-fold dilution | klm | 6.78936 | 28 | S-Sor | 1000-fold dilution | jkl | 9.74824 |
| 11 | R-Lor | 100-fold dilution | ijkl | 10.40631 | 29 | S-Ceb | undiluted | n | 0 |
| 12 | R-Lor | 1000-fold dilution | hijk | 12.44181 | 30 | S-Ceb | 10-fold dilution | ef | 20.80454 |
| 13 | B-Sor | undiluted | n | 0 | 31 | S-Ceb | 100-fold dilution | de | 24.88011 |
| 14 | B-Sor | 10-fold dilution | fghi | 16.13852 | 32 | S-Ceb | 1000-fold dilution | klm | 7.34802 |
| 15 | B-Sor | 100-fold dilution | lmn | 5.13131 | 33 | S-Lor | undiluted | n | 0 |
| 16 | B-Sor | 1000-fold dilution | mn | 0.92593 | 34 | S-Lor | 10-fold dilution | lmn | 5.49860 |
| 17 | B-Ceb | undiluted | n | 0 | 35 | S-Lor | 100-fold dilution | hijk | 12.40353 |
| 18 | B-Ceb | 10-fold dilution | c | 30.87896 | 36 | S-Lor | 1000-fold dilution | fgh | 16.92138 |

**S2B Table *O. aegyptiaca* seeds germination induced by methanol extracts of three cultivars lily below-organs at leaf-expanding growth stage.**

| Below-organs methanol extracts | | | | | | | | | |
| --- | --- | --- | --- | --- | --- | --- | --- | --- | --- |
| N | Sample | Concentration | Difference | Mean | N | Sample | Concentration | Difference | Mean |
| 1 | R-Sor | undiluted | jklmn | 19.09885 | 19 | B-Ceb | 100-fold dilution | fgh | 31.55871 |
| 2 | R-Sor | 10-fold dilution | efg | 32.33419 | 20 | B-Ceb | 1000-fold dilution | ghijk | 24.64664 |
| 3 | R-Sor | 100-fold dilution | pqr | 8.10674 | 21 | B-Lor | undiluted | r | 0 |
| 4 | R-Sor | 1000-fold dilution | r | 0 | 22 | B-Lor | 10-fold dilution | b | 60.10721 |
| 5 | R-Ceb | undiluted | r | 0 | 23 | B-Lor | 100-fold dilution | ef | 33.90514 |
| 6 | R-Ceb | 10-fold dilution | fghi | 31.06475 | 24 | B-Lor | 1000-fold dilution | mnop | 14.07348 |
| 7 | R-Ceb | 100-fold dilution | hijkl | 23.73781 | 25 | S-Sor | undiluted | qr | .92754 |
| 8 | R-Ceb | 1000-fold dilution | hijkl | 23.34467 | 26 | S-Sor | 10-fold dilution | c | 50.11342 |
| 9 | R-Lor | undiluted | r | 0 | 27 | S-Sor | 100-fold dilution | mnop | 13.60297 |
| 10 | R-Lor | 10-fold dilution | op | 9.50720 | 28 | S-Sor | 1000-fold dilution | pqr | 7.40018 |
| 11 | R-Lor | 100-fold dilution | ijkl | 22.95957 | 29 | S-Ceb | undiluted | r | 0 |
| 12 | R-Lor | 1000-fold dilution | klmno | 16.80145 | 30 | S-Ceb | 10-fold dilution | ef | 34.88121 |
| 13 | B-Sor | undiluted | r | 0 | 31 | S-Ceb | 100-fold dilution | fghij | 27.46730 |
| 14 | B-Sor | 10-fold dilution | de | 40.13665 | 32 | S-Ceb | 1000-fold dilution | jklmn | 19.00806 |
| 15 | B-Sor | 100-fold dilution | lmnop | 15.79552 | 33 | S-Lor | undiluted | r | 0 |
| 16 | B-Sor | 1000-fold dilution | nop | 10.57017 | 34 | S-Lor | 10-fold dilution | opq | 8.82896 |
| 17 | B-Ceb | undiluted | r | 0 | 35 | S-Lor | 100-fold dilution | jklm | 20.14999 |
| 18 | B-Ceb | 10-fold dilution | cd | 45.08193 | 36 | S-Lor | 1000-fold dilution | jklm | 19.52350 |

Abbreviations: R-Sor, root extracts of Sorbone; R-Ceb, root extracts of Ceb Dazzle; R-Lor, root extracts of *L.formolongo*.; B-Sor, bulb extracts of Sorbone; B-Ceb, bulb extracts of Ceb Dazzle; B-Lor, bulb extracts of *L.formolongo*.; S-Sor, scale leaf extracts of Sorbone; S-Ceb, scale leaf extracts of Ceb Dazzle; S-Lor, scale

leaf extracts of *L.formolongo*.

**S2C Table *O. aegyptiaca* seeds germination induced by aqueous extracts of three cultivars lily above-organs at leaf-expanding growth stage.**

| Above-organs aqueous extracts | | | | | | | | | |
| --- | --- | --- | --- | --- | --- | --- | --- | --- | --- |
| N | Sample | Concentration | Difference | Mean | N | Sample | Concentration | Difference | Mean |
| 1 | P-Sor | undiluted | g | 0 | 19 | A-Ceb | 100-fold dilution | c | 17.98821 |
| 2 | P-Sor | 10-fold dilution | fg | 0.95238 | 20 | A-Ceb | 1000-fold dilution | e | 7.40926 |
| 3 | P-Sor | 100-fold dilution | efg | 2.96581 | 21 | A-Lor | undiluted | g | 0 |
| 4 | P-Sor | 1000-fold dilution | g | 0 | 22 | A-Lor | 10-fold dilution | fg | 1.55556 |
| 5 | P-Ceb | undiluted | fg | 0.61728 | 23 | A-Lor | 100-fold dilution | efg | 4.16871 |
| 6 | P-Ceb | 10-fold dilution | fg | 1.82643 | 24 | A-Lor | 1000-fold dilution | fg | 1.16809 |
| 7 | P-Ceb | 100-fold dilution | fg | 2.05598 | 25 | L-Sor | undiluted | g | 0 |
| 8 | P-Ceb | 1000-fold dilution | g | 0 | 26 | L-Sor | 10-fold dilution | g | 0 |
| 9 | P-Lor | undiluted | g | 0 | 27 | L-Sor | 100-fold dilution | g | 0 |
| 10 | P-Lor | 10-fold dilution | efg | 3.81024 | 28 | L-Sor | 1000-fold dilution | g | 0 |
| 11 | P-Lor | 100-fold dilution | ef | 5.29982 | 29 | L-Ceb | undiluted | g | 0 |
| 12 | P-Lor | 1000-fold dilution | efg | 3.99932 | 30 | L-Ceb | 10-fold dilution | g | 0 |
| 13 | A-Sor | undiluted | g | 0 | 31 | L-Ceb | 100-fold dilution | g | 0 |
| 14 | A-Sor | 10-fold dilution | b | 23.31344 | 32 | L-Ceb | 1000-fold dilution | g | 0 |
| 15 | A-Sor | 100-fold dilution | b | 23.54948 | 33 | L-Lor | undiluted | g | 0 |
| 16 | A-Sor | 1000-fold dilution | d | 13.60591 | 34 | L-Lor | 10-fold dilution | g | 0 |
| 17 | A-Ceb | undiluted | g | 0 | 35 | L-Lor | 100-fold dilution | g | 0 |
| 18 | A-Ceb | 10-fold dilution | b | 22.66276 | 36 | L-Lor | 1000-fold dilution | g | 0 |

**S2D Table *O. aegyptiaca* seeds germination induced by methanol extracts of three cultivars lily above-organs at leaf-expanding growth stage.**

| Above-organs methanol extracts | | | | | | | | | |
| --- | --- | --- | --- | --- | --- | --- | --- | --- | --- |
| N | Sample | Concentration | Difference | Mean | N | Sample | Concentration | Difference | Mean |
| 1 | P-Sor | undiluted | bc | 33.7437 | 19 | A-Ceb | 100-fold dilution | b | 36.1832 |
| 2 | P-Sor | 10-fold dilution | hi | 15.7927 | 20 | A-Ceb | 1000-fold dilution | def | 25.3962 |
| 3 | P-Sor | 100-fold dilution | kl | 4.0312 | 21 | A-Lor | undiluted | l | 0 |
| 4 | P-Sor | 1000-fold dilution | l | 1.1494 | 22 | A-Lor | 10-fold dilution | ijk | 11.6252 |
| 5 | P-Ceb | undiluted | kl | 3.4119 | 23 | A-Lor | 100-fold dilution | defg | 24.0941 |
| 6 | P-Ceb | 10-fold dilution | jkl | 7.8431 | 24 | A-Lor | 1000-fold dilution | efgh | 22.7970 |
| 7 | P-Ceb | 100-fold dilution | defg | 24.7299 | 25 | L-Sor | undiluted | l | 0 |
| 8 | P-Ceb | 1000-fold dilution | cdef | 26.8296 | 26 | L-Sor | 10-fold dilution | ijk | 10.6310 |
| 9 | P-Lor | undiluted | l | 0 | 27 | L-Sor | 100-fold dilution | ijk | 10.9625 |
| 10 | P-Lor | 10-fold dilution | jkl | 6.1394 | 28 | L-Sor | 1000-fold dilution | jkl | 5.0636 |
| 11 | P-Lor | 100-fold dilution | fgh | 20.6421 | 29 | L-Ceb | undiluted | l | 0 |
| 12 | P-Lor | 1000-fold dilution | bcd | 31.9005 | 30 | L-Ceb | 10-fold dilution | ij | 12.3356 |
| 13 | A-Sor | undiluted | l | 0 | 31 | L-Ceb | 100-fold dilution | kl | 3.3774 |
| 14 | A-Sor | 10-fold dilution | bcde | 29.4034 | 32 | L-Ceb | 1000-fold dilution | kl | 3.4921 |
| 15 | A-Sor | 100-fold dilution | defg | 24.6443 | 33 | L-Lor | undiluted | l | .0000 |
| 16 | A-Sor | 1000-fold dilution | ghi | 16.9735 | 34 | L-Lor | 10-fold dilution | jkl | 5.4946 |
| 17 | A-Ceb | undiluted | l | 0 | 35 | L-Lor | 100-fold dilution | ijk | 9.6671 |
| 18 | A-Ceb | 10-fold dilution | bcde | 30.2721 | 36 | L-Lor | 1000-fold dilution | jkl | 5.6007 |

Abbreviations: P-Sor, prop root extracts of Sorbone; P-Ceb, prop root extracts of Ceb Dazzle; P-Lor, prop root extracts of *L.formolongo*.; A-Sor, aerial stem extracts of Sorbone; A-Ceb, aerial stem extracts of Ceb Dazzle; A-Lor, aerial stem extracts of *L.formolongo*.; L-Sor, leaf extracts of Sorbone; L-Ceb, leaf extracts of Ceb Dazzle; L-Lor, leaf extracts of *L.formolongo*.
