# Supplementary material for: Lily Cultivars Have Allelopathic Potential in Controlling Orobanche aegyptiaca Persoon
Source: PLoS One. 2015 Nov 13;10(11):e0142811. doi: 10.1371/journal.pone.0142811 (PMC4643976; doi:10.1371/journal.pone.0142811)
Supplement: S3 Table — (DOCX) [file pone.0142811.s003.docx]

**S3A Table *O. aegyptiaca* seeds germination induced by aqueous extracts of three cultivars lily below-organs at flowering growth stage.**

| Below-organs aqueous | | | | | | | | | |
| --- | --- | --- | --- | --- | --- | --- | --- | --- | --- |
| N | Sample | Concentration | Difference | Mean | N | Sample | Concentration | Difference | Mean |
| 1 | R-Sor | undiluted | h | 0 | 13 | B-Ceb | undiluted | h | 0 |
| 2 | R-Sor | 10-fold dilution | fg | 7.184521 | 14 | B-Ceb | 10-fold dilution | gh | 3.932665 |
| 3 | R-Sor | 100-fold dilution | ef | 9.566954 | 15 | B-Ceb | 100-fold dilution | ef | 8.982022 |
| 4 | R-Sor | 1000-fold dilution | fg | 5.343010 | 16 | B-Ceb | 1000-fold dilution | de | 9.804994 |
| 5 | R-Ceb | undiluted | h | 0 | 17 | S-Sor | undiluted | fg | 8.933826 |
| 6 | R-Ceb | 10-fold dilution | gh | 5.926426 | 18 | S-Sor | 10-fold dilution | bc | 7.875711 |
| 7 | R-Ceb | 100-fold dilution | ef | 7.907554 | 19 | S-Sor | 100-fold dilution | b | 6.735297 |
| 8 | R-Ceb | 1000-fold dilution | ef | 6.939469 | 20 | S-Sor | 1000-fold dilution | bc | 10.338805 |
| 9 | B-Sor | undiluted | h | 0 | 21 | S-Ceb | undiluted | h | 0 |
| 10 | B-Sor | 10-fold dilution | ef | 9.266351 | 22 | S-Ceb | 10-fold dilution | cd | 11.435794 |
| 11 | B-Sor | 100-fold dilution | de | 7.286154 | 23 | S-Ceb | 100-fold dilution | b | 15.327435 |
| 12 | B-Sor | 1000-fold dilution | ef | 9.859065 | 24 | S-Ceb | 1000-fold dilution | bc | 14.502212 |

**S3B Table *O. aegyptiaca* seeds germination induced by methanol extracts of three cultivars lily below-organs at flowering growth stage.**

| Below-organs methanol extracts | | | | | | | | | |
| --- | --- | --- | --- | --- | --- | --- | --- | --- | --- |
| N | Sample | Concentration | Difference | Mean | N | Sample | Concentration | Difference | Mean |
| 1 | R-Sor | undiluted | fgh | 15.14296 | 13 | B-Ceb | undiluted | fgh | 12.95275 |
| 2 | R-Sor | 10-fold dilution | fgh | 14.92883 | 14 | B-Ceb | 10-fold dilution | cd | 29.78166 |
| 3 | R-Sor | 100-fold dilution | gh | 11.37742 | 15 | B-Ceb | 100-fold dilution | cd | 29.69624 |
| 4 | R-Sor | 1000-fold dilution | hi | 5.77599 | 16 | B-Ceb | 1000-fold dilution | def | 22.70940 |
| 5 | R-Ceb | undiluted | i | 0 | 17 | S-Sor | undiluted | i | 0 |
| 6 | R-Ceb | 10-fold dilution | def | 21.94964 | 18 | S-Sor | 10-fold dilution | b | 48.36966 |
| 7 | R-Ceb | 100-fold dilution | def | 21.49224 | 19 | S-Sor | 100-fold dilution | cd | 30.42120 |
| 8 | R-Ceb | 1000-fold dilution | gh | 10.87895 | 20 | S-Sor | 1000-fold dilution | def | 21.74311 |
| 9 | B-Sor | undiluted | i | 0 | 21 | S-Ceb | undiluted | i | 0 |
| 10 | B-Sor | 10-fold dilution | c | 34.16986 | 22 | S-Ceb | 10-fold dilution | b | 47.33191 |
| 11 | B-Sor | 100-fold dilution | cd | 29.73839 | 23 | S-Ceb | 100-fold dilution | cde | 27.37118 |
| 12 | B-Sor | 1000-fold dilution | cde | 26.45868 | 24 | S-Ceb | 1000-fold dilution | efg | 18.50960 |

Abbreviations: R-Sor, root extracts of Sorbone; R-Ceb, root extracts of Ceb Dazzle; B-Sor, bulb extracts of Sorbone; B-Ceb, bulb extracts of Ceb Dazzle; S-Sor, scale leaf extracts of Sorbone; S-Ceb, scale leaf extracts of Ceb Dazzle.

**S3C Table O. aegyptiaca seeds germination induced by aqueous extracts of three cultivars lily above-organs at flowering growth stage.**

| Above-organs aqueous extracts | | | | | | | | | |
| --- | --- | --- | --- | --- | --- | --- | --- | --- | --- |
| N | Sample | Concentration | Difference | Mean | N | Sample | Concentration | Difference | Mean |
| 1 | P-Sor | undiluted | j | 0 | 19 | A-Ceb | 100-fold dilution | gh | 8.39658 |
| 2 | P-Sor | 10-fold dilution | cde | 16.59016 | 20 | A-Ceb | 1000-fold dilution | efgh | 11.79525 |
| 3 | P-Sor | 100-fold dilution | bcd | 20.08657 | 21 | A-Lor | undiluted | j | 0 |
| 4 | P-Sor | 1000-fold dilution | cde | 17.50647 | 22 | A-Lor | 10-fold dilution | def | 14.99582 |
| 5 | P-Ceb | undiluted | j | 0 | 23 | A-Lor | 100-fold dilution | b | 24.53734 |
| 6 | P-Ceb | 10-fold dilution | fgh | 10.13655 | 24 | A-Lor | 1000-fold dilution | cd | 18.41595 |
| 7 | P-Ceb | 100-fold dilution | defg | 14.03129 | 25 | L-Sor | undiluted | j | 0 |
| 8 | P-Ceb | 1000-fold dilution | bc | 22.10965 | 26 | L-Sor | 10-fold dilution | j | 0 |
| 9 | P-Lor | undiluted | ij | 2.24467 | 27 | L-Sor | 100-fold dilution | j | 0 |
| 10 | P-Lor | 10-fold dilution | def | 15.43311 | 28 | L-Sor | 1000-fold dilution | j | 0 |
| 11 | P-Lor | 100-fold dilution | cdef | 15.83193 | 29 | L-Ceb | undiluted | j | 0 |
| 12 | P-Lor | 1000-fold dilution | efgh | 11.86233 | 30 | L-Ceb | 10-fold dilution | j | 0 |
| 13 | A-Sor | undiluted | j | 0 | 31 | L-Ceb | 100-fold dilution | j | 0 |
| 14 | A-Sor | 10-fold dilution | cde | 16.73186 | 32 | L-Ceb | 1000-fold dilution | j | 0 |
| 15 | A-Sor | 100-fold dilution | cd | 18.54986 | 33 | L-Lor | undiluted | j | 0 |
| 16 | A-Sor | 1000-fold dilution | cde | 17.14767 | 34 | L-Lor | 10-fold dilution | ij | 2.32159 |
| 17 | A-Ceb | undiluted | j | 0 | 35 | L-Lor | 100-fold dilution | efgh | 11.63221 |
| 18 | A-Ceb | 10-fold dilution | hi | 6.63586 | 36 | L-Lor | 1000-fold dilution | hi | 7.24540 |

**S3D Table O. aegyptiaca seeds germination induced by methanol extracts of three cultivars lily above-organs at flowering growth stage.**

| Above-organs methanol extracts | | | | | | | | | |
| --- | --- | --- | --- | --- | --- | --- | --- | --- | --- |
| N | Sample | Concentration | Difference | Mean | N | Sample | Concentration | Difference | Mean |
| 1 | P-Sor | undiluted | ijkl | 10.45536 | 19 | A-Ceb | 100-fold dilution | hijk | 14.03569 |
| 2 | P-Sor | 10-fold dilution | cde | 28.71816 | 20 | A-Ceb | 1000-fold dilution | jklm | 7.35555 |
| 3 | P-Sor | 100-fold dilution | gh | 20.04936 | 21 | A-Lor | undiluted | m | .00000 |
| 4 | P-Sor | 1000-fold dilution | hij | 14.83069 | 22 | A-Lor | 10-fold dilution | c | 33.39964 |
| 5 | P-Ceb | undiluted | m | .00000 | 23 | A-Lor | 100-fold dilution | cd | 31.96732 |
| 6 | P-Ceb | 10-fold dilution | ijkl | 11.07809 | 24 | A-Lor | 1000-fold dilution | gh | 19.00828 |
| 7 | P-Ceb | 100-fold dilution | defg | 25.46709 | 25 | L-Sor | undiluted | lm | 4.49735 |
| 8 | P-Ceb | 1000-fold dilution | c | 35.86683 | 26 | L-Sor | 10-fold dilution | klm | 6.78312 |
| 9 | P-Lor | undiluted | gh | 20.33435 | 27 | L-Sor | 100-fold dilution | klm | 6.63901 |
| 10 | P-Lor | 10-fold dilution | defg | 25.31370 | 28 | L-Sor | 1000-fold dilution | ijkl | 9.83876 |
| 11 | P-Lor | 100-fold dilution | fgh | 20.83661 | 29 | L-Ceb | undiluted | m | .00000 |
| 12 | P-Lor | 1000-fold dilution | gh | 18.86522 | 30 | L-Ceb | 10-fold dilution | ijkl | 10.30663 |
| 13 | A-Sor | undiluted | m | .00000 | 31 | L-Ceb | 100-fold dilution | lm | 5.47419 |
| 14 | A-Sor | 10-fold dilution | efgh | 21.91939 | 32 | L-Ceb | 1000-fold dilution | lm | 3.28520 |
| 15 | A-Sor | 100-fold dilution | hi | 16.69343 | 33 | L-Lor | undiluted | defg | 25.42825 |
| 16 | A-Sor | 1000-fold dilution | hij | 14.89101 | 34 | L-Lor | 10-fold dilution | b | 43.03321 |
| 17 | A-Ceb | undiluted | m | .00000 | 35 | L-Lor | 100-fold dilution | c | 33.74045 |
| 18 | A-Ceb | 10-fold dilution | gh | 19.47583 | 36 | L-Lor | 1000-fold dilution | cdef | 28.22894 |

Abbreviations: P-Sor, prop root extracts of Sorbone; P-Ceb, prop root extracts of Ceb Dazzle; P-Lor, prop root extracts of *L.formolongo*.; A-Sor, aerial stem extracts of Sorbone; A-Ceb, aerial stem extracts of Ceb Dazzle; A-Lor, aerial stem extracts of *L.formolongo*.; L-Sor, leaf extracts of Sorbone; L-Ceb, leaf extracts of Ceb Dazzle; L-Lor, leaf extracts of *L.formolongo*.
