# Supplementary material for: Lily Cultivars Have Allelopathic Potential in Controlling Orobanche aegyptiaca Persoon
Source: PLoS One. 2015 Nov 13;10(11):e0142811. doi: 10.1371/journal.pone.0142811 (PMC4643976; doi:10.1371/journal.pone.0142811)
Supplement: S4 Table — (DOCX) [file pone.0142811.s004.docx]

**S4A Table *O. aegyptiaca* seeds germination induced by aqueous extracts of three cultivars lily below-organs at bulblet weight increasing growth stage.**

| Below-organs aqueous | | | | | | | | | |
| --- | --- | --- | --- | --- | --- | --- | --- | --- | --- |
| N | Sample | Concentration | Difference | Mean | N | Sample | Concentration | Difference | Mean |
| 1 | R-Sor | undiluted | g | 0 | 13 | B-Ceb | undiluted | g | 0 |
| 2 | R-Sor | 10-fold dilution | efg | 6.95790 | 14 | B-Ceb | 10-fold dilution | g | 0 |
| 3 | R-Sor | 100-fold dilution | cd | 16.72844 | 15 | B-Ceb | 100-fold dilution | efg | 6.89199 |
| 4 | R-Sor | 1000-fold dilution | cd | 15.21625 | 16 | B-Ceb | 1000-fold dilution | def | 8.60844 |
| 5 | R-Ceb | undiluted | g | 0 | 17 | S-Sor | undiluted | g | 0 |
| 6 | R-Ceb | 10-fold dilution | fg | 4.75425 | 18 | S-Sor | 10-fold dilution | b | 34.74640 |
| 7 | R-Ceb | 100-fold dilution | cdef | 11.05476 | 19 | S-Sor | 100-fold dilution | b | 36.04376 |
| 8 | R-Ceb | 1000-fold dilution | cd | 16.82000 | 20 | S-Sor | 1000-fold dilution | c | 18.00988 |
| 9 | B-Sor | undiluted | g | 0 | 21 | S-Ceb | undiluted | g | 0 |
| 10 | B-Sor | 10-fold dilution | cde | 13.40822 | 22 | S-Ceb | 10-fold dilution | b | 35.74574 |
| 11 | B-Sor | 100-fold dilution | cde | 14.76648 | 23 | S-Ceb | 100-fold dilution | b | 36.69165 |
| 12 | B-Sor | 1000-fold dilution | cde | 14.38375 | 24 | S-Ceb | 1000-fold dilution | b | 33.41155 |

**S4B Table *O. aegyptiaca* seeds germination induced by methanol extracts of three cultivars lily below-organs at bulblet weight increasing growth stage.**

| Below-organs methanol extracts | | | | | | | | | |
| --- | --- | --- | --- | --- | --- | --- | --- | --- | --- |
| N | Sample | Concentration | Difference | Mean | N | Sample | Concentration | Difference | Mean |
| 1 | R-Sor | undiluted | ij | 3.30534 | 13 | B-Ceb | undiluted | j | 0 |
| 2 | R-Sor | 10-fold dilution | cd | 37.70527 | 14 | B-Ceb | 10-fold dilution | de | 31.27097 |
| 3 | R-Sor | 100-fold dilution | fgh | 17.79663 | 15 | B-Ceb | 100-fold dilution | fgh | 16.83474 |
| 4 | R-Sor | 1000-fold dilution | ghi | 13.28098 | 16 | B-Ceb | 1000-fold dilution | hi | 11.19177 |
| 5 | R-Ceb | undiluted | j | 0 | 17 | S-Sor | undiluted | j | 0 |
| 6 | R-Ceb | 10-fold dilution | ghi | 13.37475 | 18 | S-Sor | 10-fold dilution | b | 56.01852 |
| 7 | R-Ceb | 100-fold dilution | de | 33.12172 | 19 | S-Sor | 100-fold dilution | de | 31.67805 |
| 8 | R-Ceb | 1000-fold dilution | de | 28.28861 | 20 | S-Sor | 1000-fold dilution | ef | 25.09152 |
| 9 | B-Sor | undiluted | j | 0 | 21 | S-Ceb | undiluted | c | 43.73540 |
| 10 | B-Sor | 10-fold dilution | hi | 10.05092 | 22 | S-Ceb | 10-fold dilution | c | 45.97301 |
| 11 | B-Sor | 100-fold dilution | efg | 23.33455 | 23 | S-Ceb | 100-fold dilution | cd | 38.11677 |
| 12 | B-Sor | 1000-fold dilution | fgh | 17.99468 | 24 | S-Ceb | 1000-fold dilution | de | 29.94330 |

Abbreviations: R-Sor, root extracts of Sorbone; R-Ceb, root extracts of Ceb Dazzle; B-Sor, bulb extracts of Sorbone; B-Ceb, bulb extracts of Ceb Dazzle; S-Sor, scale leaf extracts of Sorbone; S-Ceb, scale leaf extracts of Ceb Dazzle.

**S4C Table *O. aegyptiaca* seeds germination induced by aqueous extracts of three cultivars lily above-organs at bulblet weight increasing growth stage.**

| Above-organs aqueous extracts | | | | | | | | | |
| --- | --- | --- | --- | --- | --- | --- | --- | --- | --- |
| N | Sample | Concentration | Difference | Mean | N | Sample | Concentration | Difference | Mean |
| 1 | P-Sor | undiluted | j | 0 | 19 | A-Ceb | 100-fold dilution | ij | 0.92754 |
| 2 | P-Sor | 10-fold dilution | ghi | 10.11773 | 20 | A-Ceb | 1000-fold dilution | ij | 1.05820 |
| 3 | P-Sor | 100-fold dilution | gh | 10.85250 | 21 | A-Lor | undiluted | j | 0 |
| 4 | P-Sor | 1000-fold dilution | gh | 10.69812 | 22 | A-Lor | 10-fold dilution | ef | 21.18446 |
| 5 | P-Ceb | undiluted | j | 0 | 23 | A-Lor | 100-fold dilution | d | 39.86963 |
| 6 | P-Ceb | 10-fold dilution | hij | 2.84361 | 24 | A-Lor | 1000-fold dilution | d | 37.38716 |
| 7 | P-Ceb | 100-fold dilution | ghij | 6.15432 | 25 | L-Sor | undiluted | j | 0 |
| 8 | P-Ceb | 1000-fold dilution | hij | 2.15649 | 26 | L-Sor | 10-fold dilution | j | 0 |
| 9 | P-Lor | undiluted | ij | 1.14719 | 27 | L-Sor | 100-fold dilution | j | 0 |
| 10 | P-Lor | 10-fold dilution | fg | 14.50080 | 28 | L-Sor | 1000-fold dilution | j | 0 |
| 11 | P-Lor | 100-fold dilution | e | 26.56584 | 29 | L-Ceb | undiluted | j | 0 |
| 12 | P-Lor | 1000-fold dilution | ef | 20.76577 | 30 | L-Ceb | 10-fold dilution | j | 0 |
| 13 | A-Sor | undiluted | j | 0 | 31 | L-Ceb | 100-fold dilution | j | 0 |
| 14 | A-Sor | 10-fold dilution | j | 0 | 32 | L-Ceb | 1000-fold dilution | j | 0 |
| 15 | A-Sor | 100-fold dilution | j | 0 | 33 | L-Lor | undiluted | j | 0 |
| 16 | A-Sor | 1000-fold dilution | j | 0 | 34 | L-Lor | 10-fold dilution | d | 45.47880 |
| 17 | A-Ceb | undiluted | j | 0 | 35 | L-Lor | 100-fold dilution | c | 66.94096 |
| 18 | A-Ceb | 10-fold dilution | ij | 0.58480 | 36 | L-Lor | 1000-fold dilution | b | 76.66184 |

**S4D Table *O. aegyptiaca* seeds germination induced by methanol extracts of three cultivars lily above-organs at bulblet weight increasing growth stage.**

| Above-organs methanol extracts | | | | | | | | | |
| --- | --- | --- | --- | --- | --- | --- | --- | --- | --- |
| N | Sample | Concentration | Difference | Mean | N | Sample | Concentration | Difference | Mean |
| 1 | P-Sor | undiluted | ghijk | 10.45536 | 19 | A-Ceb | 100-fold dilution | fghi | 12.98568 |
| 2 | P-Sor | 10-fold dilution | b | 28.71816 | 20 | A-Ceb | 1000-fold dilution | ghijk | 10.36645 |
| 3 | P-Sor | 100-fold dilution | cde | 20.04936 | 21 | A-Lor | undiluted | n | 0 |
| 4 | P-Sor | 1000-fold dilution | efgh | 14.83069 | 22 | A-Lor | 10-fold dilution | def | 17.07646 |
| 5 | P-Ceb | undiluted | n | 0 | 23 | A-Lor | 100-fold dilution | ghijk | 9.68139 |
| 6 | P-Ceb | 10-fold dilution | mn | 1.62023 | 24 | A-Lor | 1000-fold dilution | ghij | 10.66892 |
| 7 | P-Ceb | 100-fold dilution | cdef | 17.88071 | 25 | L-Sor | undiluted | ijklm | 6.89672 |
| 8 | P-Ceb | 1000-fold dilution | bc | 23.44037 | 26 | L-Sor | 10-fold dilution | ijklm | 7.01933 |
| 9 | P-Lor | undiluted | n | 0 | 27 | L-Sor | 100-fold dilution | klmn | 4.12798 |
| 10 | P-Lor | 10-fold dilution | defgh | 15.19458 | 28 | L-Sor | 1000-fold dilution | lmn | 2.87919 |
| 11 | P-Lor | 100-fold dilution | fghi | 11.51071 | 29 | L-Ceb | undiluted | jklmn | 4.58333 |
| 12 | P-Lor | 1000-fold dilution | hijkl | 8.63885 | 30 | L-Ceb | 10-fold dilution | cd | 21.09428 |
| 13 | A-Sor | undiluted | n | 0 | 31 | L-Ceb | 100-fold dilution | defg | 15.70718 |
| 14 | A-Sor | 10-fold dilution | lmn | 3.17059 | 32 | L-Ceb | 1000-fold dilution | fghi | 11.76046 |
| 15 | A-Sor | 100-fold dilution | mn | 1.08985 | 33 | L-Lor | undiluted | n | 0 |
| 16 | A-Sor | 1000-fold dilution | n | 0.37037 | 34 | L-Lor | 10-fold dilution | ghijk | 10.03982 |
| 17 | A-Ceb | undiluted | n | 0 | 35 | L-Lor | 100-fold dilution | fghi | 12.13255 |
| 18 | A-Ceb | 10-fold dilution | ghijk | 9.73895 | 36 | L-Lor | 1000-fold dilution | lmn | 2.91935 |

Abbreviations: P-Sor, prop root extracts of Sorbone; P-Ceb, prop root extracts of Ceb Dazzle; P-Lor, prop root extracts of *L.formolongo*.; A-Sor, aerial stem extracts of Sorbone; A-Ceb, aerial stem extracts of Ceb Dazzle; A-Lor, aerial stem extracts of *L.formolongo*.; L-Sor, leaf extracts of Sorbone; L-Ceb, leaf extracts of Ceb Dazzle; L-Lor, leaf extracts of *L.formolongo*.
