# Supplementary material for: Lily Cultivars Have Allelopathic Potential in Controlling Orobanche aegyptiaca Persoon
Source: PLoS One. 2015 Nov 13;10(11):e0142811. doi: 10.1371/journal.pone.0142811 (PMC4643976; doi:10.1371/journal.pone.0142811)
Supplement: S5 Table — (DOCX) [file pone.0142811.s005.docx]

**S5A Table *O. aegyptiaca* seeds germination induced by aqueous extracts of lily Sorbone rhizosphere soil at different growth stage.**

| Sorbone aqueous extracts | | | | | | | | | |
| --- | --- | --- | --- | --- | --- | --- | --- | --- | --- |
| N | Sample | Concentration | Difference | Mean | N | Sample | Concentration | Difference | Mean |
| 1 | Sprouting | undiluted | hi | 4.01368 | 9 | Flowering | undiluted | fg | 13.13361 |
| 2 | Sprouting | 10-fold dilution | efg | 16.57108 | 10 | Flowering | 10-fold dilution | c | 31.45083 |
| 3 | Sprouting | 100-fold dilution | efg | 17.53690 | 11 | Flowering | 100-fold dilution | cd | 29.77425 |
| 4 | Sprouting | 1000-fold dilution | ef | 19.95157 | 12 | Flowering | 1000-fold dilution | b | 40.04216 |
| 5 | Leaf-  expanding | undiluted | i | 1.44928 | 13 | Bulblet weight icreasing | undiluted | i | 0.42735 |
| 6 | Leaf-  expanding | 10-fold dilution | efg | 15.60570 | 14 | Bulblet weight icreasing | 10-fold dilution | hi | 2.65397 |
| 7 | Leaf-  expanding | 100-fold dilution | de | 22.31589 | 15 | Bulblet weight icreasing | 100-fold dilution | gh | 9.52569 |
| 8 | Leaf-  expanding | 1000-fold dilution | cd | 27.82968 | 16 | Bulblet weight icreasing | 1000-fold dilution | g | 11.63106 |

**S5B Table *O. aegyptiaca* seeds germination induced by methanol extracts of lily Sorbone rhizosphere soil at different growth stage.**

| Sorbone methanol extracts | | | | | | | | | |
| --- | --- | --- | --- | --- | --- | --- | --- | --- | --- |
| N | Sample | Concentration | Difference | Mean | N | Sample | Concentration | Difference | Mean |
| 1 | Sprouting | undiluted | f | 0 | 9 | Flowering | undiluted | f | 0.48309 |
| 2 | Sprouting | 10-fold dilution | f | 0.55556 | 10 | Flowering | 10-fold dilution | c | 21.58507 |
| 3 | Sprouting | 100-fold dilution | ef | 3.51852 | 11 | Flowering | 100-fold dilution | b | 37.50024 |
| 4 | Sprouting | 1000-fold dilution | de | 10.23589 | 12 | Flowering | 1000-fold dilution | b | 37.74313 |
| 5 | Leaf-  expanding | undiluted | f | 0 | 13 | Bulblet weight icreasing | undiluted | f | 0 |
| 6 | Leaf-  expanding | 10-fold dilution | cd | 15.38440 | 14 | Bulblet weight icreasing | 10-fold dilution | f | 0.44444 |
| 7 | Leaf-  expanding | 100-fold dilution | c | 21.21870 | 15 | Bulblet weight icreasing | 100-fold dilution | cd | 12.88243 |
| 8 | Leaf-  expanding | 1000-fold dilution | b | 30.86640 | 16 | Bulblet weight icreasing | 1000-fold dilution | cd | 18.26110 |

**S5C Table *O. aegyptiaca* seeds germination induced by aqueous extracts of lily Ceb Dazzle rhizosphere soil at different growth stage.**

| Ceb Dazzle aqueous extracts | | | | | | | | | |
| --- | --- | --- | --- | --- | --- | --- | --- | --- | --- |
| N | Sample | Concentration | Difference | Mean | N | Sample | Concentration | Difference | Mean |
| 1 | Sprouting | undiluted | ef | 5.71821 | 9 | Flowering | undiluted | f | 2.91703 |
| 2 | Sprouting | 10-fold dilution | de | 12.80621 | 10 | Flowering | 10-fold dilution | d | 20.63696 |
| 3 | Sprouting | 100-fold dilution | d | 16.69037 | 11 | Flowering | 100-fold dilution | c | 30.49317 |
| 4 | Sprouting | 1000-fold dilution | d | 18.29545 | 12 | Flowering | 1000-fold dilution | b | 38.05708 |
| 5 | Leaf-expanding | undiluted | f | 3.04012 | 13 | Bulblet weight icreasing | undiluted | f | 0.42735 |
| 6 | Leaf-expanding | 10-fold dilution | d | 19.99636 | 14 | Bulblet weight icreasing | 10-fold dilution | f | 3.85470 |
| 7 | Leaf-expanding | 100-fold dilution | c | 28.94106 | 15 | Bulblet weight icreasing | 100-fold dilution | ef | 6.29878 |
| 8 | Leaf-expanding | 1000-fold dilution | c | 28.89628 | 16 | Bulblet weight icreasing | 1000-fold dilution | d | 18.37284 |

**S5D Table *O. aegyptiaca* seeds germination induced by methanol extracts of lily Ceb Dazzle rhizosphere soil at different growth stage.**

| Ceb Dazzle methanol extracts | | | | | | | | | |
| --- | --- | --- | --- | --- | --- | --- | --- | --- | --- |
| N | Sample | Concentration | Difference | Mean | N | Sample | Concentration | Difference | Mean |
| 1 | Sprouting | undiluted | gh | 10 | 9 | Flowering | undiluted | gh | 0.33670 |
| 2 | Sprouting | 10-fold dilution | fgh | 2.15773 | 10 | Flowering | 10-fold dilution | efg | 6.74926 |
| 3 | Sprouting | 100-fold dilution | efgh | 5.52758 | 11 | Flowering | 100-fold dilution | b | 33.62315 |
| 4 | Sprouting | 1000-fold dilution | cd | 16.83652 | 12 | Flowering | 1000-fold dilution | b | 38.24804 |
| 5 | Leaf-expanding | undiluted | h | 0 | 13 | Bulblet weight icreasing | undiluted | fgh | 1.70940 |
| 6 | Leaf-expanding | 10-fold dilution | fgh | 2.79645 | 14 | Bulblet weight icreasing | 10-fold dilution | gh | 1.05820 |
| 7 | Leaf-expanding | 100-fold dilution | de | 11.14396 | 15 | Bulblet weight icreasing | 100-fold dilution | ef | 7.99825 |
| 8 | Leaf-expanding | 1000-fold dilution | c | 17.65692 | 16 | Bulblet weight icreasing | 1000-fold dilution | cd | 16.49930 |

**S5E Table *O. aegyptiaca* seeds germination induced by aqueous extracts of lily *L.formolongo* rhizosphere soil at different growth stage.**

| *L.formolongo* aqueous extracts | | | | | | | | | |
| --- | --- | --- | --- | --- | --- | --- | --- | --- | --- |
| N | Sample | Concentration | Difference | Mean | N | Sample | Concentration | Difference | Mean |
| 1 | Sprouting | undiluted | fgh | 2.97467 | 9 | Flowering | undiluted | h | 0 |
| 2 | Sprouting | 10-fold dilution | def | 9.44545 | 10 | Flowering | 10-fold dilution | efg | 6.25886 |
| 3 | Sprouting | 100-fold dilution | defg | 8.60316 | 11 | Flowering | 100-fold dilution | de | 12.32827 |
| 4 | Sprouting | 1000-fold dilution | defg | 8.18841 | 12 | Flowering | 1000-fold dilution | c | 19.63284 |
| 5 | Leaf-expanding | undiluted | defg | 7.38407 | 13 | Bulblet weight icreasing | undiluted | h | 0 |
| 6 | Leaf-expanding | 10-fold dilution | c | 24.47088 | 14 | Bulblet weight icreasing | 10-fold dilution | gh | 2.37610 |
| 7 | Leaf-expanding | 100-fold dilution | b | 32.95560 | 15 | Bulblet weight icreasing | 100-fold dilution | d | 12.87421 |
| 8 | Leaf-expanding | 1000-fold dilution | b | 32.71985 | 16 | Bulblet weight icreasing | 1000-fold dilution | c | 19.45465 |

**S5F Table *O. aegyptiaca* seeds germination induced by methanol extracts of lily *L.formolongo* rhizosphere soil at different growth stage.**

| *L.formolongo* methanol extracts | | | | | | | | | |
| --- | --- | --- | --- | --- | --- | --- | --- | --- | --- |
| N | Sample | Concentration | Difference | Mean | N | Sample | Concentration | Difference | Mean |
| 1 | Sprouting | undiluted | e | 0 | 9 | Flowering | undiluted | e | 0 |
| 2 | Sprouting | 10-fold dilution | d | 10.76538 | 10 | Flowering | 10-fold dilution | e | 0 |
| 3 | Sprouting | 100-fold dilution | d | 16.79244 | 11 | Flowering | 100-fold dilution | e | 4.08383 |
| 4 | Sprouting | 1000-fold dilution | c | 24.15127 | 12 | Flowering | 1000-fold dilution | e | 1.81965 |
| 5 | Leaf-expanding | undiluted | e | 0 | 13 | Bulblet weight icreasing | undiluted | e | 1.51515 |
| 6 | Leaf-expanding | 10-fold dilution | d | 13.78042 | 14 | Bulblet weight icreasing | 10-fold dilution | e | 2.03891 |
| 7 | Leaf-expanding | 100-fold dilution | b | 37.28350 | 15 | Bulblet weight icreasing | 100-fold dilution | e | 2.28074 |
| 8 | Leaf-expanding | 1000-fold dilution | b | 39.66647 | 16 | Bulblet weight icreasing | 1000-fold dilution | e | 2.97485 |
